# A conceptual framework for simultaneous optimization of integrated climate scenario data and phenology models

**DOI:** 10.1007/s13593-026-01091-0

**Published:** 2026-03-12

**Authors:** Flavian Tschurr, Sven Kotlarski, Pierre Martre, Achim Walter, Lukas Roth

**Affiliations:** 1 Department of Environmental System Science, ETH Zurich, Universitätsstrasse 2, Zurich, 8092 Switzerland; 2 Federal Office of Meteorology and Climatology MeteoSwiss, Zurich, 8058 Switzerland; 3 LEPSE, Université Montpellier, INRAE, Institut Agro Montpellier, Montpellier, France

**Keywords:** Crop environment interaction, Crop growth modelling, Climate change impact, Wheat phenology, Climate scenario data, Uncertainty assessment

## Abstract

Climate-resilient crop varieties are exceedingly important for global food security. High-accuracy predictions of crop phenology in future climate scenarios require a more precise description of putative crop-environment interactions. While the accuracy of these predictions is affected by three primary sources of uncertainty — the phenology model, the climate scenario data, and their interaction — commonly used approaches consider only the phenology model as a source of uncertainty, while uncertainties arising from climate scenario data and their interaction with the model are commonly neglected. To address this gap, we developed a novel dynamic crop phenology model framework that simultaneously integrates uncertainties arising from all three sources. This model framework was developed using a large dataset of winter wheat phenology ratings and environmental covariate observations, encompassing more than 2500 locations spanning more than 80 years. It incorporates and combines up to seven environmental covariates in an additive manner, resulting in a large set of possible models from which to select. For each model, the phenological development follows a defined response curve per environmental covariate. Using climate scenario projections in Switzerland, our proposed effective model selection process helps to find the best model that reduces uncertainties in predicting future crop phenology. In addition to predictions, the model can pinpoint the driving environmental covariates for different phenological phases. Four major phenological stages are predicted for an independent subset of the large dataset and future climate scenario projections. Predictions for future climate scenario projections indicate a 22-day earlier heading for 2070–2099 compared to the reference period (1981–2010). Ignoring uncertainties arising from climate data results in contradictory predictions. These findings underscore the importance of considering all three uncertainty sources — phenology model, climate scenario data, and their interaction — in accurately predicting phenology under future climates.

**Supplementary Information:**

The online version contains supplementary material available at 10.1007/s13593-026-01091-0.

## Introduction

**Fig. 1:**
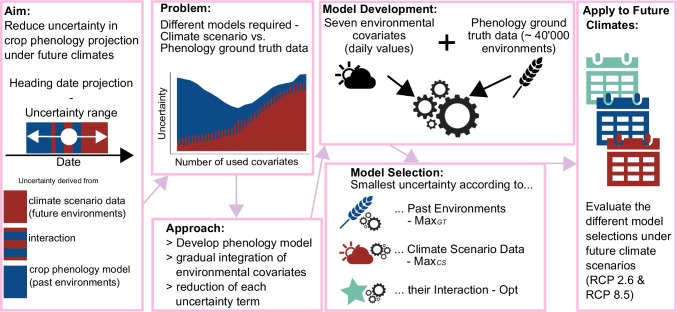
The aim of this study is to reduce the overall uncertainty in crop phenology projection under future climates (Aim). The problem faced in doing this is the different sources of uncertainty (Problem). To solve this issue, the suggested approach is to develop a novel phenology model that addresses and reduces each uncertainty term (Approach). The model was developed using seven environmental covariates and $\sim \!40'000$ ground truth phenology observations (Model Development). Then, for each uncertainty term, the best model combination is selected (Model Selection) and applied to future climate scenario data (Apply to Future Climates).

Worldwide, the primary source of human daily calorie intake relies on a handful of arable crops, such as wheat, corn, and rice (Shewry and Hey 2015). However, ongoing climate change threatens crop yield on a global scale (Jägermeyr et al. 2021). As the world’s population continues to grow and demand for food increases, these trends underscore the urgent need for solutions that can help increase crop production (Ray et al. 2012). The need for action is further emphasized by projections indicating a potential decline in future crop production and quality due to rising temperatures (Asseng et al. 2015, 2019; Rezaei et al. 2023; Guo et al. 2024).

Projections of climate change’s impact on crop production require a sound understanding of crop-environment interactions, particularly crop phenology. Different modeling approaches have been employed to predict phenology. While the simplest ones rely on only one environmental covariate, for example, growing degree days (GDD) that is based on temperature (McMaster and Wilhelm 1997), more intricate ones incorporate multiple covariates, for example, temperature and day length (Wang and Engel 1998). Recent studies use machine learning methodologies to integrate large numbers of covariates (Guo et al. 2021). Phenology models are trained on data from the past. Typically, the more environmental covariates are included, the lower the error (or uncertainty; see Sect. 3.1) becomes before increasing again due to overfitting. In contrast to this, climate scenario data is intended to project the future development of several environmental covariates: the more covariates shall be further used, the higher their uncertainty will be.

Phenology and climate scenario modeling approaches are used to identify future climate stressors and their impact on crop growth, as well as to determine optimized crop phenology for future climates (Semenov et al. 2014; Stratonovitch and Semenov 2015; Rogger et al. 2021). For critical phenology phases, abiotic stresses such as heat or drought impose significant crop yield penalties. The abundance and severity of such stresses are expected to increase in the future (Trnka et al. 2014; Mickelbart et al. 2015). Crops ripening in early summer will minimize the risk of severe mid-summer drought stress reducing their yield (Shavrukov et al. 2017; Yashavanthakumar et al. 2021; Dorrani-Nejad et al. 2022). On the other hand, earliness implies the disadvantage of a shortened growing season. Consequently, yield would be reduced, as shown by ideotype studies that have identified a more extended grain-filling phase as crucial for yield improvement (Semenov et al. 2014; Senapati and Semenov 2020; Senapati et al. 2022). Phenology prediction models with adequate accuracy provide a way to find an optimal trade-off between stress avoidance and optimized phenology phase duration for future climate scenarios. Such measures to robustly assess crop production risks caused by climatic changes are urgently needed (Holzkämper 2017; Kim et al. 2025).

**Fig. 2:**
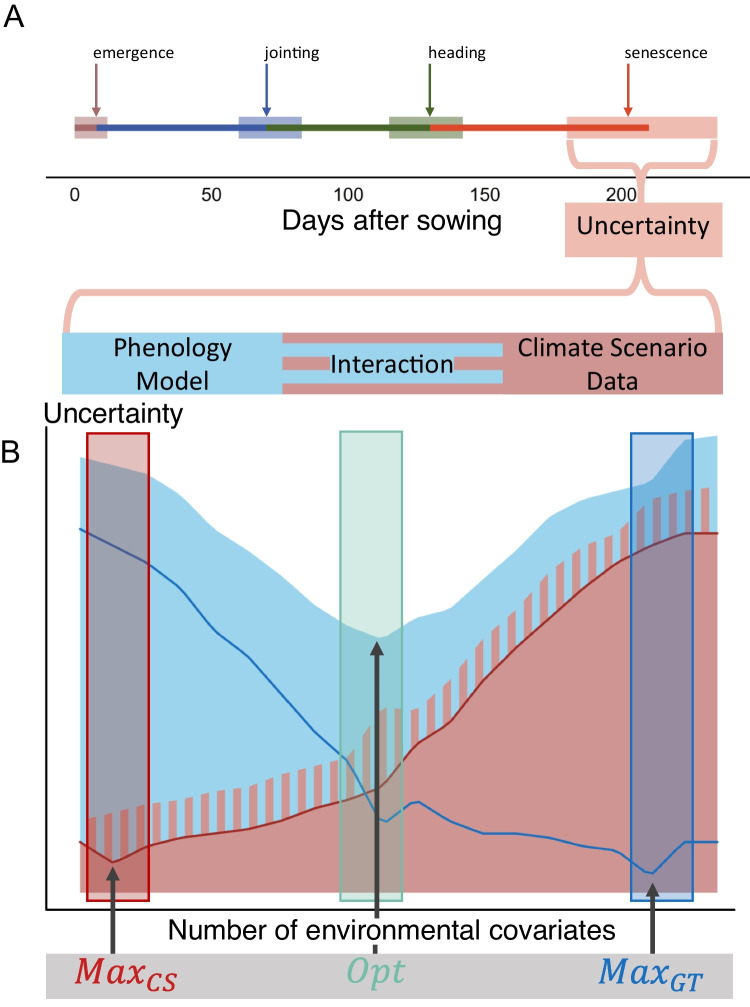
Assumed projections of phenological stages (**A**) for future climates are subject to three different sources of uncertainty (**B**): the uncertainty (or error) deriving from the phenology model (blue), from the climate scenario data (red), and their interaction (hatched), schematically represented. To reduce uncertainty in the phenology model, as many available and informative environmental covariates as possible are used (blue rectangle, $Max_{GT}$). For the climate scenario data, environmental covariates are expected to provide less uncertainty for output sets with fewer covariates (red rectangle, $Max_{CS}$). Finding the optimal model selection represents a trade-off between these two extremes (green rectangle, *Opt*). Note: The lines in (**B**) represent the individual uncertainty from one of the different sources, and the colored areas are the corresponding cumulative uncertainty.

The projection of phenology for future climate scenarios faces three sources of uncertainty: (1) the phenology model, (2) the climate scenario data, and (3) the interaction between the phenology model and the climate scenario data (Fig. 1 and 2) (Tschurr et al. 2020). Over time, crop models have evolved by incorporating additional parameters and covariates in an effort to improve accuracy (Wang et al. 2017). The same applies to climate models (Knutti and Sedláček 2013; Smith et al. 2020). It has been found that using more complex output sets (i.e., multiple projected climate variables) of climate scenario data tends to increase the model spread, as different climate models and downscaling processes handle certain processes differently. The contradiction of using as many environmental covariates as possible (to increase the accuracy of the phenology model) while simultaneously using as few as possible (to reduce the uncertainty deriving from the climate scenario data) suggests that an optimum model exists that minimizes the overall combined error (Fig. 2).

Finding such optimized models requires a model framework with the flexibility to vary the number of included covariates. Previous crop models (e.g., (van Diepen et al. 1989; Wang and Engel 1998; Jamieson et al. 1998; Brown et al. 2018; Roth, Barendregt, et al. 2022) suggest different functions describing growth responses to environmental covariates. However, these models have not been designed to flexibly vary the number of input variables but to rather predict plant growth as accurately as possible.

To provide the flexibility to vary the number of covariates, we propose a crop phenology model framework that uses additive response curves to environmental covariates, an approach inspired by Pasley et al. (2022). Response functions to various environmental covariates can be incrementally incorporated, allowing for the optimal — most overall uncertainty-reducing — combination. Such an optimization then leads to a selection of which environmental covariates should be included or excluded from a final model.

Different datasets are required to quantify the sources of uncertainty. The error (or uncertainty) of the phenology model itself requires ground truth phenology ratings, with which predicted model values can be compared to actual observed values. The uncertainty of the climate scenario data can be examined by applying the model framework to a reference period with available environmental observations and climate projections. Evaluating models solely based on ground truth data or climate scenario data will result in differing model selection results compared to simultaneously integrating both sources of uncertainty. These differences allow for the evaluating of a third source of uncertainty, the interaction effect between the phenology model and the climate scenario projection.

The proposed framework thus allows for the quantitative evaluation of uncertainties originating from environmental covariates-driven phenology and climate scenario projection models. On the downside, the proposed model framework does not account for other factors, such as soil type, management decisions, or genotype specificity. In addition, the framework’s purely additive nature prevents modeling the interaction effects of environmental covariates and the inclusion of growth-related feedback loops. However, the flexibility of such an additive framework to vary the number of environmental covariates is a prerequisite for simultaneously optimizing phenology model and climate scenario projection errors, which outweighs the disadvantages in the context of our specific research question.

In summary, this study aims (i) to develop a phenology model framework with the required flexibility to vary the number of environmental covariates, (ii) to assess the uncertainty originating from the introduced model framework, climate scenario data, and their interaction, by using a reference period with ground truth phenology data, climate observations, and climate scenario projections, and (iii) to apply the winning model to future climate scenarios to derive trends in phenology.

## Data sources and preparation

Winter wheat was used as a model crop in this study. As ground truth, and hence the base of the model framework, we used up to seven environmental covariates (daily minimal temperature ($^{\circ }$C)(tasmin), daily mean temperature ($^{\circ }$C) (tas), daily maximal temperature ($^{\circ }$C) (tasmax), daily average relative humidity (%) (RH), precipitation (mm/day) (pr) transformed to standardized precipitation index (SPI), daily total global radiation (J/cm^2^) (GR) and air vapor pressure deficit (hPa) (VPD)) in daily granularity on up to 40,000 year$\times$location combinations (environments). These environments cover entire Germany with more than 2500 locations throughout more than 80 years (1936 to 2022) (Fig. A.1). Dates of sowing, emergence, jointing, heading, senescence, and environmental covariate observations are available.

For Germany’s neighboring country, Switzerland, climate scenario data is available: The Swiss National Centre for Climate Services (NCCS) provides climate scenario projections for the future, including 12–31 individual simulations per scenario to capture uncertainties of environmental covariates (CH2018 2018; Fischer et al. 2022). These data were used to assess the uncertainty arising from the climate scenario data itself on phenology projections in future climates.

**Fig. 3:**
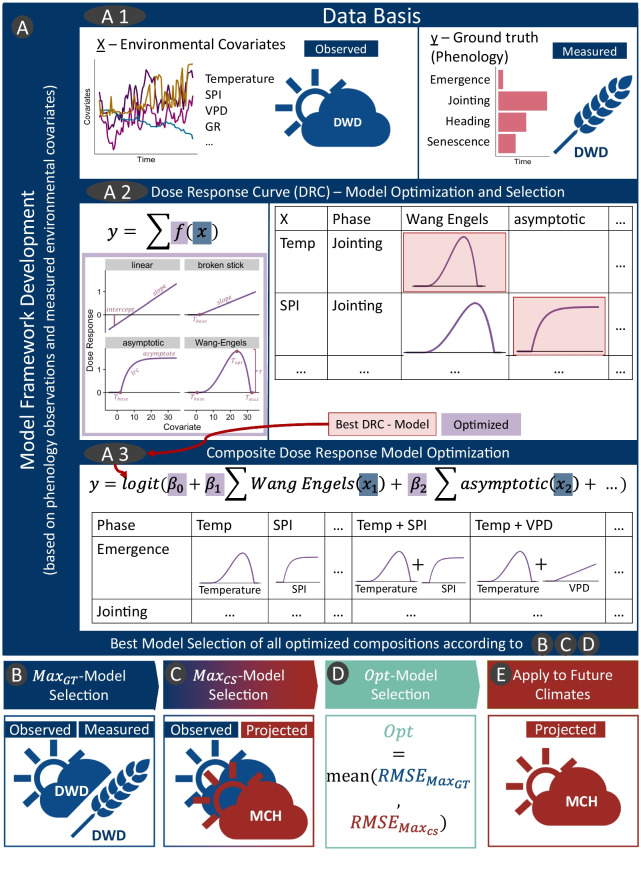
Overview of proposed model optimization process. The process starts with a dose-response curve (DRC) calibration for individual environmental covariates and growth phases using observational data (**A**, derived from the German weather service (DWD)). This calibration is followed by one of three possible model selection strategies: model selection based on the ground truth data ($Max_{GT}$; (**B**)), model selection based on the climate scenario data ($Max_{CS}$; derived from MeteoSwiss (MCH); (**C**)), and model selection based on the best combination of both (*Opt*; (**D**)) using the root mean squared error (RMSE).

In the following section, the data used in this study are described (Fig. 3A1); then, in the subsequent section, the model framework is specified (Fig. 3A2-3); and finally the model selection processes (Fig. 3B-D) are explained.

### Phenology data

Winter wheat phenological stages and environmental data were obtained from the open data server of DeutscherWetter Dienst (DWD) (https://opendata.dwd.de) (Fig. 3A1). DWD maintains a uniquely dense phenology observation network that collects data over the whole country from various agricultural and non-agricultural plants (Kaspar et al. 2014). Measured dates of emergence, jointing, heading, and senescence defined four major growing phases: emergence, vegetative growth (jointing), generative growth, and maturity. In total, 47,477 environments were used for the sowing to emergence period (from 3,135 stations), 41,718 for emergence to jointing (from 2,787 stations), 44,680 for jointing to heading (from 2,891 stations), and 15,532 for heading to senescence (from 2,020 stations).

To compare the predictions of the developed model framework that used no genotypic information with genotype-specific information, we utilized a second dataset. This second, genotype-specific phenology dataset was based on the public GABI wheat dataset (8 environments; 5 different locations in 2009 and 2010) (Gogna et al. 2022), combined with six additional years of wheat heading date measurements derived from the ETH Zurich field phenotyping platform “FIP” (Kirchgessner et al. 2017; Roth et al. 2025). For the GABI data, published Best linear unbiased estimates (BLUEs) were used. For the FIP data, BLUEs were calculated using a linear mixed model in SpATS with genotype and check (yes/no) as fixed, row and column as random effects, and a spatial smoothing component in row-column direction.

After spatial correction for field gradients and other interfering factors per environment, we evaluated the range of heading dates as the difference between the earliest and latest heading dates per environment. This estimate of genotype-specific variance served to put the results of the proposed genotype-agnostic models into context.

### Environmental covariates

Environmental covariates were obtained for the same DWD weather station as the phenological stages if available. For most of the monitored locations tasmin, tas, tasmax, RH, pr and GR were available. Data gaps, as well as missing stations, were imputed from the Joint Research Centre (JRC) Crop Growth Monitoring System (CGMS) gridded (25 km grid resolution) meteorological data base (Biavetti et al. 2014). The next closest grid point to the DWD station within a radius of 20 km was considered for the imputation. A correlation analysis per covariate between DWD data and JRC for overlapping stations showed a strong Pearson’s correlation coefficient of $\sim$ 0.95. Consequently, we did not apply any further interpolation modeling approach to the JRC data (see Table A.1).

Precipitation (pr) values were transformed to the 30-day SPI (McKee et al. 1993) with the R package SPEI (Beguería and Vicente-Serrano 2023). In contrast to precipitation, the SPI is continuous and has a close-to-normal distribution. The VPD was calculated based on temperature and relative humidity.

All available data (phenology ratings and corresponding environmental covariates) were split into a training data set (80 %, Fig. 3A) and a validation data set (20 %, Fig. 3B). The stratified random split in the training and validation sets allowed for equally distributed year-densities in both sets (Fig. A.2). This resulted in an equal spatial distribution in relation to the longitude of stations but a slight over-representation of lower latitudes in the training (Fig. A.3).

### CH2018 - future climate scenario data

Regarding climate projections, we employed the CH2018 Climate Scenarios for Switzerland, which are the current national reference scenarios released in 2018 (CH2018 2018; Fischer et al. 2022). CH2018 consists of a range of datasets and further products, all available via a central website (www.climate-scenarios.ch). Data at daily resolution for the period 1981–2099 are available for a large number of observational measurement sites (DAILY-LOCAL product; variables covered: daily mean/minimum/maximum 2 m-temperature, daily precipitation sum, daily mean global radiation, daily mean 10 m wind speed, daily mean near-surface relative humidity). This data was obtained by bias adjusting and downscaling the raw model output of 68 regional climate model (RCM) simulations originating from the EURO-CORDEX initiative (Kotlarski et al. 2023; Jacob et al. 2020) against site-scale and gridded surface observations in the reference period 1981–2010 (Feigenwinter et al. 2018). The 68 climate model simulations covered three different greenhouse gas emission scenarios: representative concentration pathway (RCP) 2.6, 4.5, and 8.5 (van Vuuren et al. 2011). Although bias-adjusting the climate model output partly corrected for systematic biases of the raw climate models, certain biases might still have been present, for instance, relating to temporal structures or inter-variable dependencies (Ivanov and Kotlarski 2017; Maraun 2016). It was recommended to validate and assess the usability of the CH2018 data in the reference period 1981–2010 for any given application (Ivanov and Kotlarski 2017). In the present work, we used the DAILY-LOCAL CH2018 data product for 36 stations in Switzerland, all located below 1000 m a.s.l. (Table A.5, Fig. 3C-E and A.1). The CH2018 climate scenario dataset has been shown to be suitable to quantify the impact of data dimensionality on uncertainty for Switzerland (Tschurr et al. 2020). However, there are no open phenological observation datasets for Switzerland to match these climate scenario data. Consequently, for phenology data, we preferred the dataset from DWD from the neighboring country Germany.

## Methods

We used the described data above to develop a model framework consisting of two successive optimization steps (Fig. 3 A2-A3). This was followed by three model selection steps (Fig. 3 B-D) and the application of the selected models to future climates (Fig. 3 E).

### Terminology: uncertainty and error

Uncertainty in climate sciences refers to the range of probable outcomes or the degree of confidence in projections about future climate conditions originating from complex system interactions, natural variability, and model limitations (Curry and Webster 2011; Mastrandrea et al. 2011). Error in agriculture typically refers to quantifiable deviations or inaccuracies in measurements or predictions (Lobell and Asseng 2017; Jones et al. 2017). In this work, when combining phenological predictions with climate scenario data, we use uncertainty as an umbrella term that encompasses both the broader concept of uncertainty from climate sciences and the more specific notion of error from agricultural studies, while acknowledging the nuanced differences in their original fields (Hopster 2023; Refsgaard et al. 2007).

### Model framework development

The base of the model framework was dose-response curves (DRC), functions that describe the relationship between an environmental covariate and crop development. These dose-response curves (DRCs) have free parameters that we optimized and calibrated using ground truth weather data and phenology ratings from the DWD and the JRC (Fig. 3A2). For each phenological phase and environmental covariate, we used the best performing DRC and combined them into composite models. These composite models used one up to all seven environmental covariate responses, in all potential combinations, in a general linear model (GLM) with a binomial probability distribution (logit) (Fig. 3A3),

1 $$\begin{aligned} \begin{aligned} \text {Pr}(Y_p=1 | {X})&=\text {logit}^{-1}(\eta _p)\, \\ \eta _p&= \beta _{0p} \;+\; \sum _{c \in \mathcal {C}} \delta _{c,p}\,\beta _{c,p}\,Z_{c,p},\\ Z_{c,p}&= \sum _{d=1}^{D_p} f_{c}\!\big (x_{c,d};\theta _c\big ), \end{aligned} \end{aligned}$$

where $Y_p \in \{0,1\}$ is a binary indicator that phenological phase *p* is reached ($p \in \{$ sowing–emergence, emergence–jointing, jointing–heading, heading–senescence $\}$). Its probability is modelled with a generalized linear model with a binary distribution using ${\text {logit}}^{-1}(\eta _p)$. $\eta _p$ is a linear predictor and $\beta _{0p}$ the intercept for phase *p*. $\mathcal {C}$ is a set of covariates included, $\{\text {tasmin}, \text {tas}, \text {tasmax}, \text {VPD}, \text {SPI}, \text {GR}\}$, each included at most once. $x_{c,d}$ is the observed daily value of covariate *c* on day *d* within the accumulation window $D_p$ for phase *p*. $f_c(\cdot ;\theta _c)$ is a precalibrated dose–response function for covariate *c* (linear, broken-stick, asymptotic, or Wang–Engel) with fixed parameters $\theta _c$. $Z_{c,p}$ is an accumulated dose of covariate *c* over $D_p$ days for phase *p*. $\delta _{c,p}\in \{0,1\}$ is an indicator whether covariate *c* is included for phase *p* or not. $\beta _{c,p}$ are GLM coefficients multiplying $Z_{c,p}$ when $\delta _{c,p}=1$; estimated from data.

We then employed a model selection process to identify the best composite model from the multitude of possible model assortments. Afterwards, the sub-models for each phenological phase were connected to enable the prediction of the whole phenology.

#### Dose response curves

For each of the seven environmental covariates, DRCs were fitted. The used DRCs had two to four free parameters (Table 1 and Fig. 3A2).

We chose four different DRCs in this study. The most simple DRC considers linear growth over the whole range of covariate input *x* with an intercept and a slope parameter, also allowing negative response values (Fig. 3A2),

2 $$\begin{aligned} f(x) = \text {intercept} + x \times \text {slope} \,. \end{aligned}$$

The second DRC (broken stick) has a minimal threshold ($\Psi _\text {base}$) below which the response is zero and a slope (Fig. 3A2),

3 $$\begin{aligned} f(x) = {\left\{ \begin{array}{ll} (x - \Psi _\text {base}) \times \text {slope} & , x > \Psi _\text {base} \\ 0 & , x \le \Psi _\text {base} \,. \end{array}\right. } \end{aligned}$$

**Table 1:** Dose response curve parameters and constraints used for model fitting. While $\Psi _\text {base}$ refers to the minimal value below which no response is expected, $\Psi _\text {opt}$ refers to the optimal value at which the maximal response is expected, and $\Psi _\text {max}$ refers to the maximal value above which no response is expected. In the asymptotic equation, the asymptote (Asym) follows the logarithm of a rate constant (lrc). Additionally, the Wang-Engels equation shows the parameter *r* that scales the response curve.

Type of dose-response curve	Parameters	Constraints	Equation
Linear	Intercept Slope		Eq. 2
Broken stick	$\Psi _\text {base}$ slope		Eq. 3
Asymptotic	$\Psi _\text {base}$ lrc Asym		Eq. 4
Wang-Engels	$$\Psi _\text {base}$$ $$\Psi _\text {opt}$$$$\Psi _\text {max}$$ *r*	$$\Psi _\text {base}$$ < $$\Psi _\text {opt}$$ < $$\Psi _\text {max}$$	Eq. 5 & 6

As third DRC, an asymptotic function (4) was considered (as used in (Roth et al. 2022, 2024)) (Fig. 3A2). The function describes a curve in which growth starts at a minimum covariate value ($\Psi _\text {base}$) and reaches an asymptote (Asym) following the logarithm of a rate constant (lrc),

4 $$\begin{aligned} \begin{aligned} r(x)&= \text {Asym} \times (1 - e^{-e^\text {lrc} \times (x - \Psi _\text {base})}) \\ f(x)&= {\left\{ \begin{array}{ll} r(x) & , r(x) > 0 \\ 0 & , r(x) \le 0 \,. \end{array}\right. } \end{aligned} \end{aligned}$$

The fourth applied DRC is the so-called Wang-Engels function, as developed and used in Wang and Engel (1998), that describes a non-linear response between a minimum covariate value below which growth is zero ($\Psi _\text {base}$), an optimum value ($\Psi _\text {opt}$) for which growth is maximized to *r*, and a maximum value for which growth reaches zero again ($\Psi _\text {max}$) (Fig. 3A2),

5 $$\begin{aligned} f(x) = {\left\{ \begin{array}{ll} r \times \frac{2(x - \Psi _\text {base})^{\alpha }(\Psi _\text {opt} - \Psi _\text {base})^{\alpha } - (x - T_\text {min})^{2\alpha }}{(\Psi _\text {opt} - \Psi _\text {base})^{2\alpha }} & , \Psi _\text {base}< x < \Psi _\text {max} \\ 0 & , \text {otherwise} \end{array}\right. } \end{aligned}$$

6 $$\begin{aligned} \alpha = \frac{ln(2)}{ln(\frac{(\Psi _\text {max} - \Psi _\text {min})}{(\Psi _\text {opt} - \Psi _\text {min})})} \,. \end{aligned}$$

For each covariate, responses in the form of DRCs were individually calibrated for the four phenological phases: sowing-emergence, emergence-jointing, jointing-heading, and heading-senescence. In the case of tas, tasmin, tasmax, and GR, only the Wang-Engels DRC was used. Due to the effects of climate change, it was expected that average temperatures and the frequency of extreme temperature events will increase. The Wang-Engels DRC incorporates the parameters $\Psi _\text {base}$ and $\Psi _\text {max}$, which limit the maximal development rate and thus avoid overestimation of the development rate at very low and high temperatures. Given the degree of collinearity between temperature and GR, the Wang-Engels DRC was also employed for GR. No prior assumption was made for the other covariates. Consequently, four different DRCs (linear, non-linear, asymptotic, Wang-Engels) were applied to RH, SPI, and VPD, resulting in 12 combinations per phenological phase. In addition to these 12 DRCs, the three established DRCs of the temperatures (tasmin, tas, and tasmax) and GR were used, resulting in 16 covariate-DRC combinations per phenology phase (Fig. 3A2).

#### Model calibration and validation

In the following, we set starting parameters based on the best knowledge and practice to avoid non-convergence of the optimization process or end values resulting in boundary conditions. Parameter estimation was done in a two-step process optimization using the nloptr package in R (Johnson 2007) with the auglag function. COBYLA was chosen as the local solver, with a local absolute tolerance of 10$^{-24}$ and a relative tolerance of 10$^{-2}$. In the first step, a coarse parameter optimization was done with a maximum iteration of 500 (before the optimization was stopped by the current best result). Starting parameters and boundaries were selected according to the quantile of environmental covariate inputs for parameters that are in the unit of an environmental covariate, such as $\Psi _\text {max}$, $\Psi _\text {base}$, or $\Psi _\text {opt}$, respectively, depending on prior knowledge for parameters that were not in the unit of a corresponding environmental covariate, such as slope or Asym (see Table A.2). We used quantiles of observed environmental covariates because we desired reproducible and meaningful starting values whenever possible. Choosing environmental covariate quantiles as the starting values for DRC also made our decision independent of the environmental covariates. The coarsely fitted parameters were used as starting values in the second step. The boundaries were defined as the starting value minus itself for the lower boundary and the starting value plus itself for the upper boundary, respectively. Again, the auglag function was used with a maximum iteration of 1000 times the length of the input and, as before, a local absolute tolerance of 10$^{-24}$ and relative tolerance of 10$^{-8}$ (default value). We applied this two-step approach, which used one coarser optimization step followed by another with a higher number of maximal iterations, since it reduced the overall computing time.

To increase the robustness of DRC parameter estimations, an ensemble of 20 estimations, each with a random 80 % subset of the training data (64 % of the total data per run), was created (as in Rogger et al. (2021)). We then chose the median of the ensemble as the final parameter estimation (Fig. A.4 and A.5). One DRC was selected per environmental covariate based on the highest Pearson’s (cor) score between measured and modeled phenology-phase duration.

#### Dose response curve combination resulting in multiple composite models

In the next step, we built composite models including one to all available (seven) environmental covariates to predict each phenological phase individually (Fig. 3A3). The previously calibrated DRCs were combined to calculate the cumulative response to the environmental covariates over time. For each possible combination of environmental covariates and a specific phenological phase, a GLM was fitted, which resulted in 508 individual fitted GLMs or composite models, respectively (127 per phenological phase). The decision to use a GLM was based on the fact that such models can handle collinearity but not non-linearity. The non-linear characteristics of responses were covered by the previous and covariate-independent DRC modeling step, and the GLM reused the same data set to model dependencies among covariates.

A binary response model was chosen as the prediction needed to be binary (0 to indicate that the phase was not reached yet, and 1 to indicate that the target phenological phase was reached). The optimal threshold of the combined response of multiple DRCs was derived through a Receiver Operating Characteristic (ROC) analysis with the R package pROC (Robin et al. 2011). To create a model encompassing all four phenological phases, the selected models, one for each phase, were combined consecutively, depending on the model selection outlined in the following sections.

### Model selection process

Out of this multitude of composite models, we aimed to select the ones that best reduced the different sources of uncertainty by assessing the smallest root mean squared error (RMSE) on the validation set, a common technique to prevent overfitting (Hawkins 2004). Three distinct strategies were employed for model selection: The $Max_{GT}$ selected a model based on ground truth phenology and weather data only (Fig. 3B), thereby minimizing the error (or uncertainty) deriving from the phenology model. The best model selection for the climate scenario use-case ($Max_{CS}$) selected a model based on climate scenario projections and ground truth weather data only (Fig. 3C), minimizing the uncertainty derived from the climate scenario data. The *Opt* combined the two approaches (Fig. 3D). These steps (B-D) will be referred to as the model selection process in the following. The selected models were then applied to future climate projections (Fig. 3E).

#### $Max_{GT}$ Model selection

In the first selection step, we sought the most accurate prediction of the ground truth data among the already fitted models (Sect. 3.2.3). For this, phenology phase duration predictions of models based on the previously calibrated DRCs were compared with observations (ground truth ratings of the phenology from DWD) from the independent validation dataset. Pearson’s correlation coefficient, RMSE, and mean absolute error (MAE) were calculated. The most accurate model per complexity was selected from all fitted composite models of the corresponding phenological phase, according to the smallest RMSE. The other metrics were not used for model selection but were used to interpret and evaluate the model selection process. Potential collinear parameters were handled by the GLM: Models with an increasing number of covariates ensured that for highly collinear covariates, the more predictive covariate ended up in the selected model with a reduced number of covariates, and the less predictive were added for models with a larger number of covariates. By calculating all potential combinations of covariates and selecting the best models based on an objective metric, we avoided equifinality issues and yielded deterministic results.

#### $Max_{CS}$ Model selection

To estimate and minimize the uncertainty derived from the climate scenario data, the validation period (1981–2010) for which both climate scenario projections and weather observation data were available was used. We used the previously described CH2018 dataset (climate scenario data - Sect. 2.3) and weather observation data. We applied the best model from the previous $Max_{GT}$ selection step for each complexity level (one to seven covariates) once to observed weather data and once to climate scenario projection data for a reference period (1981–2010) of climate scenario data (Fig. 3C).

For this purpose, a set of 36 stations in Switzerland, which are located in arable land (below 1000 m a.s.l.), was selected (Table A.5). By testing only the best environmental covariate combination per complexity and phenological phase from $Max_{GT}$, we maintained physiological plausibility and comparability over all model selection steps.

Differences between the projections based on observed environmental covariates and simulated environmental covariates (CH2018 data) between 1981 and 2010 were interpreted as uncertainty arising from the climate scenario data. To reduce bias deriving from the previous phenological phase, we set the beginning of each phenological phase to the median of all observed data, i.e., a fixed day of the year (Table A.6).

Selecting the best model was again based on the RMSE. To reduce potential bias deriving from RCMs, an averaging over the RCMs was done. With this approach, the effects of potential outliers and of RCMs stronger represented in the climate scenario data ensemble, respectively, were reduced (CH2018 2018). Consequently, we derived an RMSE value for each phenology model per phenological phase and selected the best model per phase based on these values. One should note that this optimization step does not consider ground truth data, and hence, may introduce a large bias. Consequently, one needs a way to integrate both selection approaches, $Max_{GT}$ and $Max_{CS}$, to optimize for both consistency of prediction with ground truth, but also reduced sensitivity to climate data projections, which is described in the following.

#### *Opt* Model selection

According to the hypothesis made in the introduction, the $Max_{GT}$ and $Max_{CS}$ model selection strategies are expected to lead to contradicting results. While the $Max_{GT}$ is expected to favor solutions with many environmental covariates, $Max_{CS}$ is expected to prefer solutions with a few environmental covariates only (Fig. 2). A combined selection strategy is required to find the optimum between the two approaches, embodying the “Goldilocks Rule” by finding the “just right” number of covariates that balances these conflicting needs for minimizing overall uncertainty.

As a straightforward solution to this need, the mean RMSE between the performance of corresponding $Max_{CS}$ and $Max_{GT}$ model pairs was calculated for each phenological phase independently. We determined then the model pair with the smallest mean RMSE within the range of complexity of the $Max_{CS}$ and the $Max_{GT}$ as the best combination, the *Opt* model (Fig. 3D).

### Uncertainty propagation in the climate scenario data

To estimate the propagated uncertainty over the combination of environmental covariates, we adopt a modified root-sums-of-squares approach. The combined error $\epsilon _f$ for a function $f = x_1 + x_2 + \dots + x_n$ is approximated as:

7 $$\begin{aligned} \epsilon _f = \sqrt{\epsilon _{x_1}^2 + \epsilon _{x_2}^2 + \dots + \epsilon _{x_n}^2} \end{aligned}$$

where $\epsilon _{x_i}$ denotes the RMSE associated with the *i*-th used environmental covariate in the model selection (x; max seven). The RMSE is derived after a min/max standardization per environmental covariate over the observation and the climate scenario data for the reference period, representing the expected uncertainty between climate scenario data and weather observation data. This method assumes errors are uncorrelated and roughly homoscedastic across phenological phases (Farrance and Frenkel 2012). This uncertainty propagation was applied to all potential environmental covariate combinations (127).

Further, we can standardize the calculated skill scores (RMSE) from the previous model selection steps with the standard deviation of the duration of a phenological phase length derived from the DWD phenology observation data. This allows us to compare the overall propagated uncertainty in the selected model.

### Application to climate scenario data

After the model selection by using three different strategies ($Max_{GT}$, $Max_{CS}$, and *Opt*), the chosen phenology models were applied to the CH2018 climate scenario data for the RCP 2.6 and RCP 8.5 pathways. We used a fixed sowing date according to Table A.6 for all model selections and scenarios. Median values per station were calculated for two 30-year periods from 2020 to 2049 (2035) and 2070 to 2099 (2085). Furthermore, the development over time was calculated by using a two-sided rolling mean over 30 years for each climate scenario output climate model simulation ensemble separately, and minima, median, and maxima values were later calculated for the stations used. As the $Max_{CS}$ selection is designed to minimize the uncertainty from the climate scenario data, but not optimized to produce accurate projections, we exclude the projections of $Max_{CS}$ in the further sections to enable a more straightforward interpretation of the model performance, and only report the below-laying trend in the appendix.

## Results and discussion

### Model development

In our work, inspired by Pasley et al. (2022), we break down the growing period into different simplified phases. In two consecutive steps, we fit a model to these phenological phases. In a first step, the DRC that the model is composed of is optimized per environmental covariate within each phenological phase. Then, in a second step, a GLM with a binomial distribution is fitted to each potential environmental covariate combination. Applying the DRCs to the environmental covariates and then using the fitted GLM allows us to predict the end of each phenological phase. We validate the model on an independent test data set of phenological observations (20% of the total available data).

#### Dose response curve calibration

The phenological phase had an evident influence on the calibrated DRC parameters (Fig. A.6). In addition, for one specific environmental covariate, different DRC performed best (in terms of correlation between modeled and measured phase duration) for different phenological phases (Table A.3). Within each phase, except sowing-emergence, one or more DRC-environmental covariate predictions showed a moderate (>0.4) to strong (>0.6) correlation coefficient (Table A.3). Very weak correlations except for VPD were found for covariates in the emergence-jointing phase. After selecting the best calibrated DRC per phenology phase and environmental covariate, DRC for different covariates were combined to GLMs including one to seven covariates, resulting in 127 different covariate combinations (Fig. 3A3).

One limitation regarding the selected DRCs is that they only model “positive development” — i.e., the continuous increase of plant biomass and progression of growth stages. Yet under harsh conditions, biomass can decrease; leaves die, and developmental progression is severely hampered. Hence, the approach does not directly account for stress events such as frost stress, which is known to affect plant development (Tschurr et al. 2023). Nevertheless, all DRCs (except for the linear) incorporate a minimal value that an environmental covariate must reach before development can occur ($\Psi _\text {base}$). Additionally, the Wang-Engels function has a maximum parameter ($\Psi _\text {max}$). These can indirectly represent stressors, however, not by negatively affecting the development, but by setting the development rate to zero. Including stress responses that prolong instead of accelerate phenology phases has the potential to improve the accuracy of the models.

#### Model validation on test dataset

**Fig. 4:**
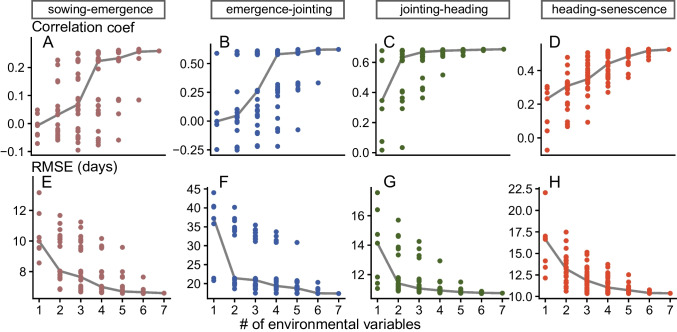
Results of the 127 different environmental covariate combinations to which the selected dose-response curves have been applied to predict the phenological phase duration with the fitted GLM for each phenological phase (different panels). The x-axis represents the number of used environmental covariates. The y-axis represents the correlation coefficient (A-D) in the upper row and the RMSE (E-H) of the model performance on the independent test dataset in the lower row. The gray line shows the median values.

We applied the 127 combinations to an independent test data set to validate the model performance on ground truth data (phenology observations). For each phenological phase, the cor and RMSE have been calculated (Fig. 4). The median cor shows for all phenological phases an increase by an increased number of environmental covariates (Fig. 4 upper row), whereas the median of the RMSE shows a decrease with an increasing number of environmental covariates (Fig. 4 lower row).

In order to assess any issues regarding a potential overfitting of the models, we compared the cor and RMSE derived from the training and test datasets. Similar or improved metrics in test scenarios refer to no overfitting (Fig. A.7) (Lever et al. 2016). Both cor and RMSE were well-aligned along the 1:1 line between training and test performance, not indicating any overfitting issue.

Then, a model selection based on the three strategies $Max_{GT}$, $Max_{CS}$, and *Opt* was performed.

### Model selection process

#### $Max_{GT}$ Model selection

**Fig. 5:**
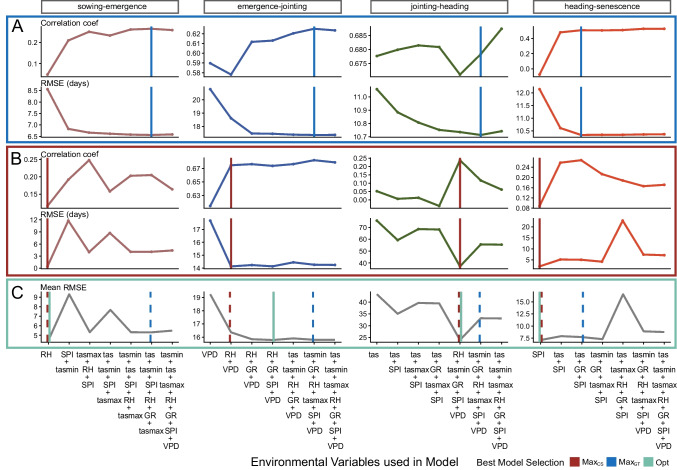
Results of the model selection process for the three different strategies $Max_{GT}$ (**A**), $Max_{CS}$ (**B**), and *Opt* (**C**) for all phenology phases (sowing-emergence, emergence-jointing, jointing-heading, and heading-senescence). For (**A**), correlation coefficients (upper row) and RMSE (lower row) between the observed phenology duration and predictions are indicated. Results are reported for increasing model complexity (x-axis) from one up to seven environmental covariates. Correlation coefficients (upper row) and RMSE (lower row) between predicted phenology phase duration based on observed and simulated climate data are indicated for B. Results are reported for increasing numbers of covariates (x-axis). For C, the mean RMSE of model pairs applied to the validation dataset (**A**) and the climate scenarios (**B**) is indicated (grey line). The vertical lines show the best model selection results for the three selection types: the “ground truth use-case” ($Max_{GT}$ - blue line), the “climate scenario use-case” (*Max* *CS* - red line), and the overall “optimal for the combined use-case” of the two (*Opt* - green line). The used environmental covariates are daily mean (tas), min (tasmin), and max (tasmax) temperature, the vapor pressure deficit (VPD), relative humidity (RH), global radiation (GR), and the standardized precipitation index (SPI).

In all phases, except the heading-senescence phase, a model selection with six environmental covariates showed the best performance (Fig. 5A). In all of these model selections, environmental covariates based on temperature (tasmin, tasmax), relative humidity (RH), radiation (GR), and precipitation (SPI) were included. The best model selection for the sowing-emergence phase additionally included tas. For emergence-jointing and jointing-heading, VPD was selected instead of tas. For the last phenological phase, a model with three environmental covariates based on temperature, radiation, and precipitation (tas, GR, and SPI) was selected according to our model selection process. In general, selecting the best model according to the lowest RMSE was in line with the highest correlation value (Fig. 5A). However, for the last phase, a model with more environmental covariates would have increased the correlation coefficient value, but would have slightly worsened the RMSE.

The correlations that were obtained varied strongly between the different phenological phases. A weak correlation was found for the sowing-emergence phase with a value of 0.27 (MAE of 3.4 days). For the other three phases, correlations were moderate (0.51, heading-senescence; MAE of 3.8 days) to strong (0.68, jointing-heading; MAE of 4.1 days) (TableA.4). In summary, the uncertainty (shown as RMSE) deriving from the $Max_{GT}$ model selection was in the range of 7 to 17 days, depending on the phenology phase.

From a physiological perspective, the $Max_{GT}$ model selection results are reasonable. The sowing-emergence phase is not only driven by environmental factors but is also severely influenced by farmers’ decisions. Sowing in unfavorable conditions can delay emergence. Nevertheless, the main factors when sowing takes place are not only environmental conditions but also the workload on a farm and the accessibility of the fields. The $Max_{GT}$ selected a model with six environmental covariates, showing that a rather complex solution is suitable if extensive ground truth data is available.

In the case of winter wheat, the emergence-jointing phase usually contains a cold period, influenced by various micro-meteorological conditions. For example, during the winter period, the fields in Germany are sometimes covered by snow. The environmental covariates are measured 2 m above ground, representing another micro-meteorology than the plants directly face. Also, the snow cover reduces or stops the potential for photosynthesis, directly influencing the development. However, with the available environmental covariates, such effects are only indirectly related to the data. This is also represented in the $Max_{GT}$ selection, favoring a model selection with six environmental covariates, covering a broad spectrum of potential micro-meteorological conditions. Hence, all available environmental covariates have been used except for tas.

Between jointing and heading is the primary phase where biomass is produced in winter wheat. Therefore, GR, water availability (SPI), and the temperature are expected to be the main driving variables. The model selection process represented this covariate preference well across the different complexity levels (Fig. 5). The final $Max_{GT}$ selection for the jointing-heading phase additionally included RH and VPD, factors known to be potential limiting factors, i.e., too high VPD representing potential adverse weather conditions during the springtime (Grossiord et al. 2020).

The senescence phase is mainly driven by water availability (SPI) and temperature (Slafer et al. 2023; Christopher et al. 2016). In the model selection process, the model with just one environmental covariate was based on SPI, and the best model based on SPI, tas, and GR (Fig. 5A). The dependence on GR may represent the potential of the plant to conduct photosynthesis, a limiting factor in the grain-filling phase.

**Precision achieved in view of the absence of genotype specificity of the model** None of the selected models, including the one suggested by $Max_{GT}$, achieved a perfect validation result on the corresponding validation dataset. This finding highlights a significant limitation of all examined models: They do not consider differences between varieties and genotypes, as this information is not available in the analyzed dataset. Despite a dataset covering approximately 40,000 environments, the lack of genotypic details remained a fundamental limitation. Across different phenology phases, RMSE values ranged from 7 to nearly 17 days. To contextualize these errors, we contrasted them with the GABI dataset (Gogna et al. 2022) and several years of wheat heading date measurements from our field station in Eschikon Switzerland (Kirchgessner et al. 2017).

The GABI+Eschikon dataset includes data from up to 381 wheat genotypes across 13 environments. The data show variety-dependent heading date variations ranging from 8.8 to 29.6 days per environment (Table A.9). Notably, these averages closely reflect the errors observed in our study (approximately eleven days for the $Max_{GT}$ based on ground truth data) (Table A.4), indicating that a large extent of remaining prediction errors may be related to cultivar-specific responses.

#### $Max_{CS}$ Model selection

According to the uncertainty propagation between the observation and climate scenario data in the reference period (1981–2010) (Fig. A.8). We expect that model selection with fewer environmental covariates will be preferred in the model selection process of $Max_{CS}$ as the propagated uncertainty increases with increasing model complexity. However, environmental covariate combinations with a higher complexity show a smaller propagated uncertainty than less complex (see Fig. A.8). In this step, we expect simpler models to be selected with one up to three or maximally four environmental covariates.

For three out of four growth phases, the model selection based on climate scenario data ($Max_{CS}$), preferred models with one or two covariates only (Fig. 5B): sowing-emergence (one covariate) with a RMSE of 0.46 days, emergence-jointing (two covariates) with a RMSE of 14.13 days, jointing-heading (five covariates) with a RMSE of 37.12 days, and heading-senescence (one covariate) with a RMSE of 2.05 days. A higher number of covariates generally increased the RMSE between the climate projections and the climate observation data in the used reference period. The correlation coefficients did not always follow this trend. For emergence-jointing (0.68) and jointing-heading (0.24), models with six or five covariates, respectively, had the highest correlation coefficients. In summary, the uncertainty (shown as RMSE) deriving from the $Max_{CS}$ model selection was in the range of 0 to 37 days, depending on the phenology phase. The very low errors achieved by simplistic models with just one covariate can be explained by their tendency to predict environment-independent average phase durations that minimize the RMSE but also the correlation coefficient.

#### *Opt* Model selection

To combine the $Max_{CS}$ and $Max_{GT}$ selection approaches, the mean RMSE between the two models per phenology phase and level of model complexity was derived (Fig. 5C). Applying the “Goldilocks Rule” to find the “just right,” or *Opt* model. For the phase emergence-jointing, the optimal covariate level complexity of *Opt* was between the one of $Max_{GT}$ and $Max_{CS}$. For all other phases, *Opt* and $Max_{CS}$ suggested similar models. In summary, the uncertainty deriving from interactions of the climate scenario projection with the chosen phenology model was negligible compared to the strong individual effects.

**Fig. 6:**
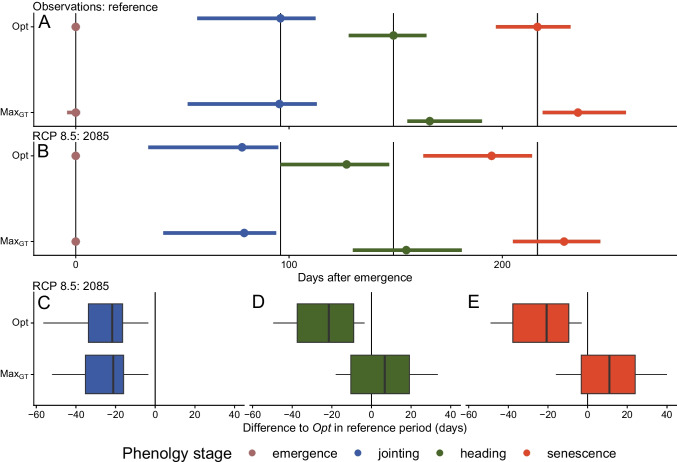
Predictions of phenology phase duration are displayed for climate scenario projections of two different periods: reference (1981–2010; measured weather data; A) and 2085 (2060–2099; B). The climate scenario (RCP 8.5) is applied for the future period. For the two periods, the different models suggested by the selection methods ($Max_{GT}$ and *Opt*) are applied. Lines in plots represent the range of phenology prediction medians per station. Points represent the median across all stations. Sowing is fixed to October 30 of each year. In the lower panel (C-E), boxplots indicate differences between the model selected by *Opt* in the reference period and the corresponding models selected by $Max_{GT}$ and *Opt* in the period 2085 with RCP 8.5 in days.

### Relative model performance and equifinality

We can compare the model selections by looking at their relative uncertainty by standardizing each with the standard deviation of the according phenological phase duration of the ground truth data of DWD (Fig. A.9). The trend of the $Max_{GT}$ and $Max_{CS}$ selections can be seen equally in Fig. 5. The *Opt* is between the $Max_{GT}$ $Max_{CS}$ selection. The emergence-jointing phase shows the lowest values overall regarding relative uncertainty. For this phase, the standard deviation of the ground truth data also shows the highest values of almost 20 days (Table A.7). Compared to the calculated propagated uncertainty of the climate scenario data (Fig. A.8), the uncertainty of the applied model selections is increased in most of the cases for the $Max_{CS}$ model.

The *Opt* selection follows in the pattern closely the $Max_{CS}$ selection. Indicating that the uncertainty stemming from the climate scenario data was more influential compared to the uncertainty that could be reduced by the $Max_{GT}$ model. This is especially visible in the jointing-heading phase by the relatively low and constant values of the $Max_{GT}$ model selection.

The fact that the models selected by the $Max_{CS}$ and *Opt* methods showed very similar propagated uncertainty but included different covariates is a sign of equifinality, where different model strategies can lead to the same output. However, as stated by Beven and Freer (2001), in forecasting models, reduction of equifinality is secondary compared to more accurate predictions. In this work, we chose a data-driven approach to test whether we can select a model optimizing the combined error from ground truth and climate scenario data. Setting specific constraints during the model selection process, such as which environmental covariates must be included or not, would have biased this overall selection. Stretching the boundaries of the parsimonity principle, in order to find the best model, many others have been necessary, and not just the most simple one (Coelho et al. 2019)

### Future climates

According to the different model selection approaches, we applied the three best phenology models to a future climate scenario dataset (CH2018 dataset, Fig. 3E), focusing on 36 weather stations in Switzerland. The prediction of dates of emergence, jointing, heading, and senescence was performed over two different periods: the reference period (1981–2010) and the projection period (2060–2099). For the projection period, the two scenarios RCP 2.6 and RCP 8.5 were chosen (Fig. 6, 7, A.11,A.12 and A.13).

**Fig. 7:**
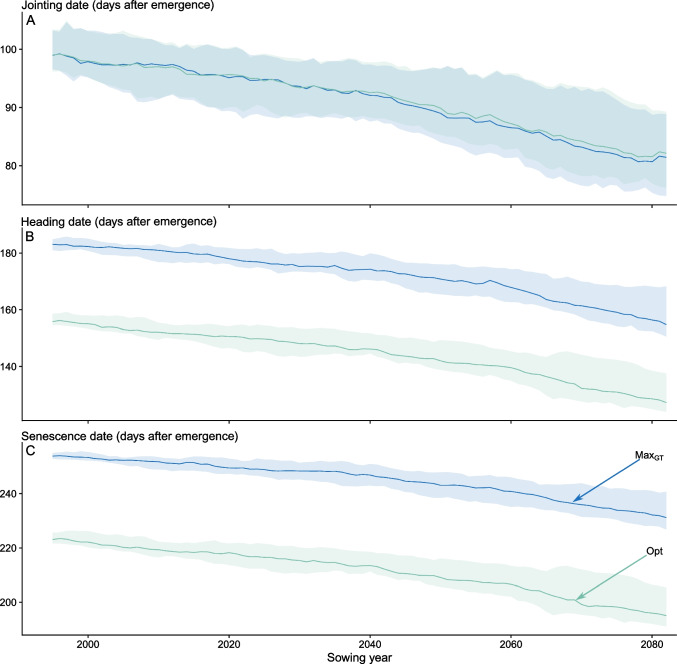
Predictions of jointing (**A**), heading (**B**), and senescence (**C**) date until 2100 for the different model selection strategies ($Max_{GT}$ and *Opt*) for the RCP 8.5 scenario at the station REH (Zurich-Reckenholz) in days after emergence (DAE). For further examples (RCP 2.6 and another example station) see Fig. A.11, A.12 and A.13).

While we see the $Max_{GT}$ as the current state of the art and the *Opt* as our optimal model selection, in line with the “Goldilocks Rule,” the $Max_{CS}$ was designed to enable selecting the *Opt* selection. Hence, the $Max_{CS}$ does not have the primary purpose to offer standalone, accurate projections. Therefore, we exclude it from the following sections to better focus on the model comparison of the $Max_{GT}$ and *Opt*.

Phenology predictions for climate scenario projections showed pronounced divergence later in the season, depending on the phenology model selection strategy, especially for heading and senescence. Conversely, the phenology predictions for jointing showed only minor dependencies on the phenology model selection strategy.

For heading dates, shifts towards earlier heading dates at the end of the next century were observed for the models suggested by *Opt*. Based on the optimized phenology prediction model *Opt*, the heading date in winter wheat will occur 22 days earlier at the end of the century compared to the reference period for RCP 8.5 (Fig. 6 and Table A.8). Contradicting, performing model selection based on ground truth data only $Max_{GT}$) suggested a phenology model that predicts — relative to the *Opt* model — a 7 days delayed heading date at the end of the century.

To compare the influence of the model selection, namely $Max_{GT}$ with the *Opt* selection, on future climate projections, the predictions of the phenological stages during the reference period of the *Opt* were taken as reference. The relative shifts compared to this reference are shown in Fig. 6 (lower panel). The data emphasize the importance of model selection by comparing the models selected by $Max_{GT}$ and *Opt*, for which the predicted shifts in heading data for RCP 8.5 concerning the reference period were offset by 28.25 days between the models (Table A.8).

Focusing on one station only (Zurich-Reckenholz (REH) under the RCP 8.5 scenario from 1981 to 2099, consistent patterns were evident (Fig. 7). As observed in the overall analysis comprising 36 stations, the differences among the models suggested by different selection methods became more pronounced as the growth period progressed, particularly from the jointing to the senescence phase (see also Figs. A.14 and A.15).

The results indicated a consistent progression towards earlier phenological stages, with a gradual acceleration in this shift continuing to the year 2100, as observed across all models. An increase in uncertainty accompanied this temporal shift.

Regardless of the model selection, a shift towards earlier phenology was observed in the results (Fig. 6 and 7). For the *Opt* model, the heading date was shifted earlier for 22 days, when the period 2085 under the RCP 8.5 scenario was compared with the reference period (Fig. A.10  and Table A.8). (He et al. 2024) reported comparable trends (11 to 14 days earlier until 2050). The projected shift can be interpreted as an escape mechanism of plants to avoid stressors, such as summer heatwaves or droughts. However, an earlier heading date also shortens the growth period of the crop, which is known to negatively affect yield (Lopes and Reynolds 2012; Montazeaud et al. 2016; Christopher et al. 2017). Interestingly, the genetic variance found in heading date in the GABI+Eschikon dataset was in the same range as the predicted shift. This could be interpreted as the current wheat breeding material (as represented in the GABI+Eschikon dataset) providing enough variability to breed cultivars optimally suited for future climate conditions.

### The importance of appropriate model selection strategies

The model selection proposed by $Max_{GT}$ led to consistent results for all phases in selecting temperature (tas, tasmin or tasmax), SPI, and GR. Temperature, radiation, and water availability are well-known drivers of phenology development (van Diepen et al. 1989; Wang and Engel 1998).

The emergence-jointing and jointing-heading phases are the two longest of the four examined phenology phases. It is expected that environmental interactions in such protracted phases are pronounced. In agreement with this hypothesis, the $Max_{GT}$ and *Opt* methods selected models with more environmental covariates during these phases compared to the other periods.

Overall, a clear and noteworthy distinction emerged among the three model selection strategies — $Max_{GT}$, $Max_{CS}$, and *Opt* (Fig. 5C, 6, and 7). This distinction can be attributed to the different optimization and selection processes. The $Max_{GT}$ was optimized and selected to perform well regarding phenology ratings (ground truth data) but not on climate scenario projections. The $Max_{CS}$ and *Opt* model selection methods included climate scenario projections in the model selection process. The clear segregation between the model selections on future climate scenario projections demonstrates the impact and importance of including not only ground truth data but also projected climate data if aiming for predictions of crop phenology related to climate scenario projections.

Previous studies categorized the influential sources of uncertainty in future yield projections as climate model, down-scaling method, or crop model derived. Down-scaling methods did not appear to be of high relevance. Still, depending on the study site, either the underlying climate model (Global Climate Model) or the crop model drove the uncertainty. Furthermore, site-specific parameters are also suggested to play a crucial role in more robust climate-crop modeling (Wang et al. 2020). The clear segregation between results of different model selections found in this work underscores the potential impact of the proposed *Opt* method in reducing the overall uncertainty associated with phenology projection in future climates.

### Limitations of the proposed model framework

This developed conceptual framework is a start to further reduce the uncertainty in phenology projections for future climate scenarios and hence serves as a preliminary or starting point for further work. We therefore want to specifically emphasize the limitations that are faced at this stage to enable further development within the scientific community as straightforwardly as possible. Future projections should be interpreted with caution, acknowledging that $Max_{CS}$ and $Max_{GT}$ are designed primarily to minimize uncertainty arising from climate scenario data or ground truth data. Their focus is not necessarily on achieving the most accurate future projections, but rather on reducing the potential uncertainty associated with these data sources.

A significant limitation is the available data for such modeling approaches. The lack of genotype specificity is a consequence of the fact that the data used does not provide this information. However, other limitations are more intrinsic to the framework, which limits its application to research questions other than the one addressed in this work. We will discuss them in the following.

The suggested model is purely additive, preventing it from including interactions between covariates. Moreover, phenology phases are not linked, which prevents cascades, like a very cold emergence-jointing phase that may also affect the jointing-heading phase.

In this work, we decided not to incorporate feedback loops such as increased growth, resulting in increased water demand, which would subsequently lead to a decrease in growth if precipitation was not sufficient. Such feedback loops would obscure the sources of uncertainty (climate scenario projections or crop model), as environmental covariates in climate scenario models are not independent (Tschurr et al. 2020). For example, to investigate the response to temperature using a model framework that considers feedback loops, we would also need to integrate precipitation information. Projected temperature and precipitation projections from climate scenarios may not be independent, consequently confounding the effects of uncertainties arising from the climate scenario data with those from the crop model. The model choice in this work was based on the requirement to dynamically include or exclude covariates to select the best covariate combination.

In this work, applying a phenology model to climate scenario projection was identified as a major source of uncertainty, with errors greater than a factor of two compared to the phenology model errors. With the proposed framework, such error (or uncertainty) sources can be identified. Once an optimal covariate selection in dependence of the climate projection data is done, switching back to conventional models, e.g., process-based crop models, could be of great advantage. Such models may be better suited to incorporate biotic and abiotic stressors that are known as yield-limiting factors (Chenu et al. 2017; Minoli et al. 2022; Singh et al. 2023). The use of process-based crop models would also allow to incorporate effects of management and soil characteristics, critical sources of variation in agricultural systems (Zhu et al. 2024) that are absent in the proposed model framework.

### Outlook

The model choice strongly influenced the results of this study. Similarly, previous studies determined crop models as an influential source for uncertainty (Xiong et al. 2019). This finding further implies that the integration of models, such as improving crop models by enriching them with climate scenario data arising from climate models, must be thoroughly considered. As climate change affects agricultural production, increasing the accuracy of such models’ projections is of utmost importance. The proposed model framework and selection process show a potential way to adapt crop models to reduce uncertainty from multiple sources. It is a crucial step to enable the usage of effective models for specific uses, such as breeding. The use of models capable of projecting the performance of a crop variety under future climate scenarios would enable the breeding of varieties better adapted to future stress scenarios. Releasing a new crop variety takes several years and is always lagging behind the effects of ongoing changes in the climate due to global warming. Therefore, gaining time with the proposed approach is crucial to shorten this time lag and to further reduce the uncertainty of the model projection, integrating genotype-specificity into the model framework.

Introducing genotype specificity is highly relevant to reduce model uncertainties (Roth et al. 2024). However, there are no large publicly available data sets comparable to the one used in this study that are genotype-specific. With the advent of publicly funded high-throughput field phenotyping (HTFP) approaches, the required genotype-specific information can be expected to be available on a much larger scale within the coming years. This will then provide genotype-specific information on crop physiology and can serve as a valuable asset in the breeding process (Challinor et al. 2016; Reynolds and Langridge 2016; Araus et al. 2018; Atkinson et al. 2018). The proposed framework can easily be applied to other crops (Tschurr et al. 2025).

Finally, modelling approaches such as the one developed in this study will be of importance to utilize the full potential of HTFP data for predictions of genotype performance in the breeding process. Insights from such DRC models could be used to integrate traditional, well-established crop growth models into genomic prediction approaches (Millet et al. 2019).

## Conclusion

In this work, we present a novel model framework that allows selecting a phenology prediction model that optimizes for uncertainties arising from the climate scenario and ground truth data simultaneously. This model predicts heading for winter wheat to occur 22 days earlier at the end of the century than at present in Switzerland. We find noteworthy differences (up to 28 days) between this model and other models that do not account for climate scenario data uncertainties, demonstrating the importance of model selection when predicting phenology based on future climate projections.

While phenology models profit from including as many meaningful environmental covariates as possible — assuming that overfitting is prevented — one should be as restrictive as possible if using climate scenario data as input. It is highly recommended to assess the uncertainty arising from applying a crop model to climate scenario projections beforehand. At a minimum, the introduced uncertainty can be evaluated using a reference period of observations and climate projections. A more optimal solution, however, is provided by the novel, dynamic modeling framework presented in this work, which actively minimizes this uncertainty by simultaneously optimizing for an optimal compromise, consistent with the “Goldilocks Rule.”

## Supplementary Information

Below is the link to the electronic supplementary material.

Supplementary file 1 (pdf 1925 KB)

## Data Availability

Phenology and weather data in Germany are derived from the DWD. Missing weather data in Germany has been filled with data from the JRC. All climate data are derived from MeteoSwiss from the CH2018 dataset. All data sources are cited correspondingly in the manuscript.

## Code availability

Code is available at: DynamicPhenoModel4ClimScen.git.
