# Supplementary material for: A conceptual framework for simultaneous optimization of integrated climate scenario data and phenology models
Source: Agron Sustain Dev. 2026 Mar 12;46(2):22. doi: 10.1007/s13593-026-01091-0 (PMC12982321; doi:10.1007/s13593-026-01091-0)
Supplement: Supplementary file 1 — (pdf 1925 KB) [file 13593_2026_1091_MOESM1_ESM.pdf]

A Supplementary Materials

A.1 Supplementary Figures

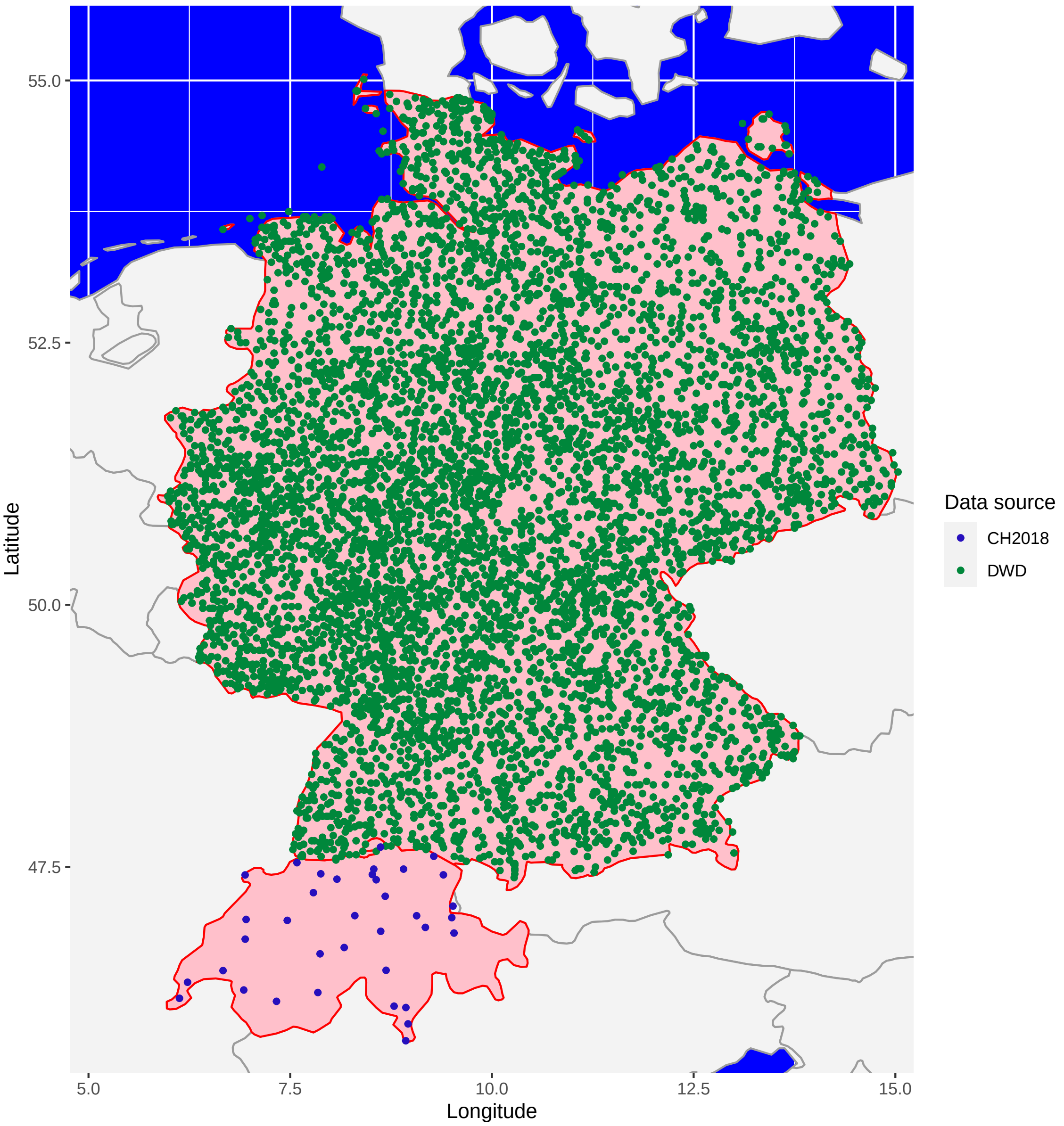

Figure A.1: Locations of the used German weather service (DWD) weather stations (green) and the CH2018 weather stations in Switzerland (blue). At the DWD stations, also phenology ratings have been gathered that were used for the DRC optimization and the Max_{GT}. The CH2018 station in Switzerland measured environmental covaraites from 1981-2010 and additionally have climate scenario data available from 1981-2010

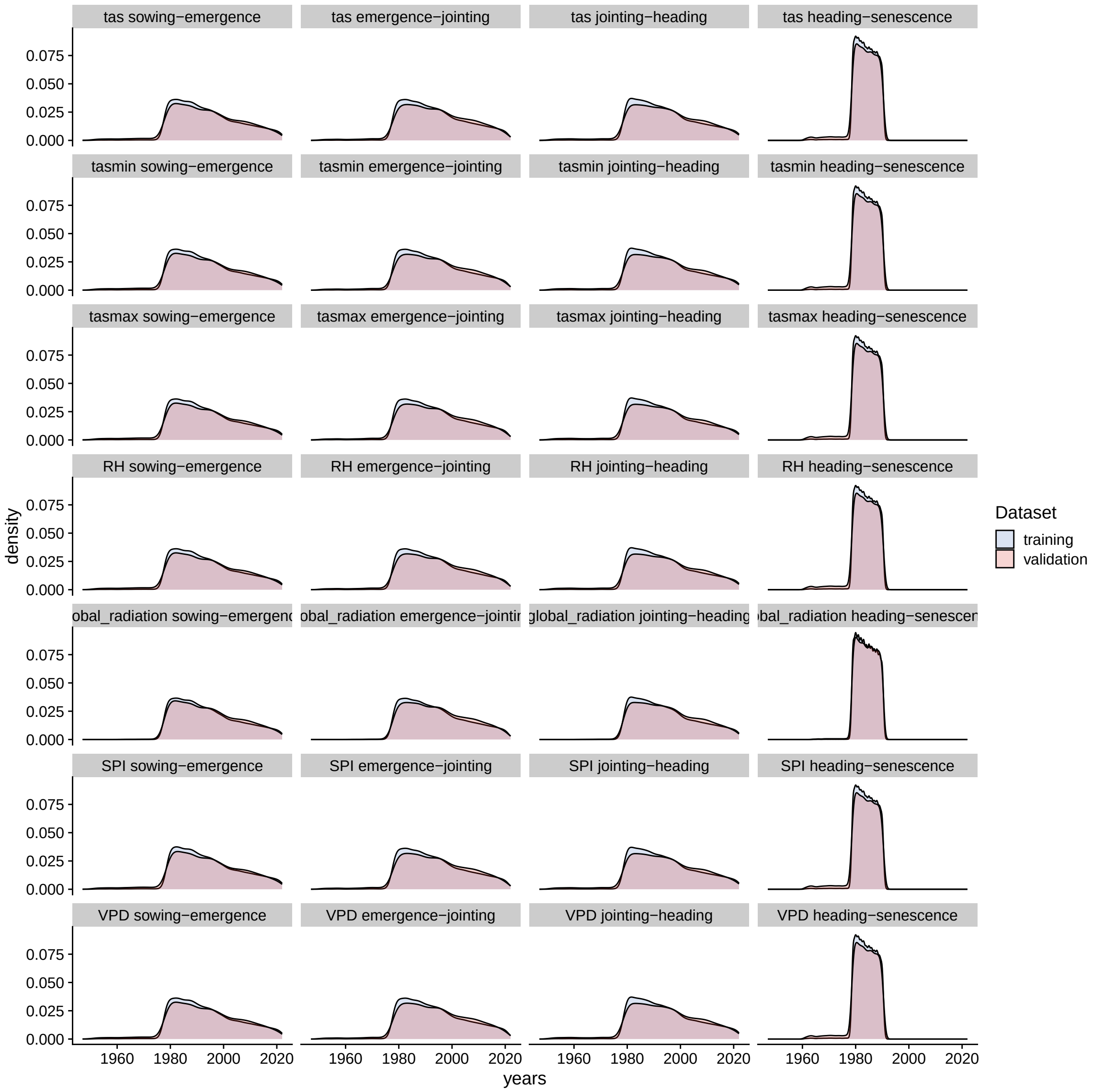

Figure A.2: Distribution of the selected years for the training and validation dataset. Each line of panels shows one covariate for the selected four phenology phases.

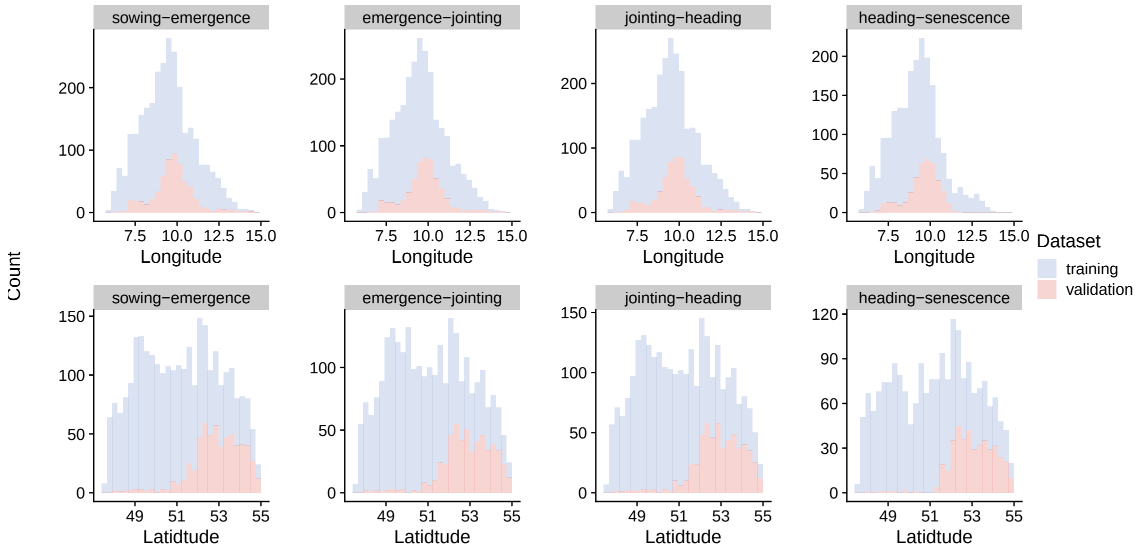

Figure A.3: Distribution of the training and validation dataset according to longitude (upper row) and latitude (lower row) for the different phenological phases (columns).

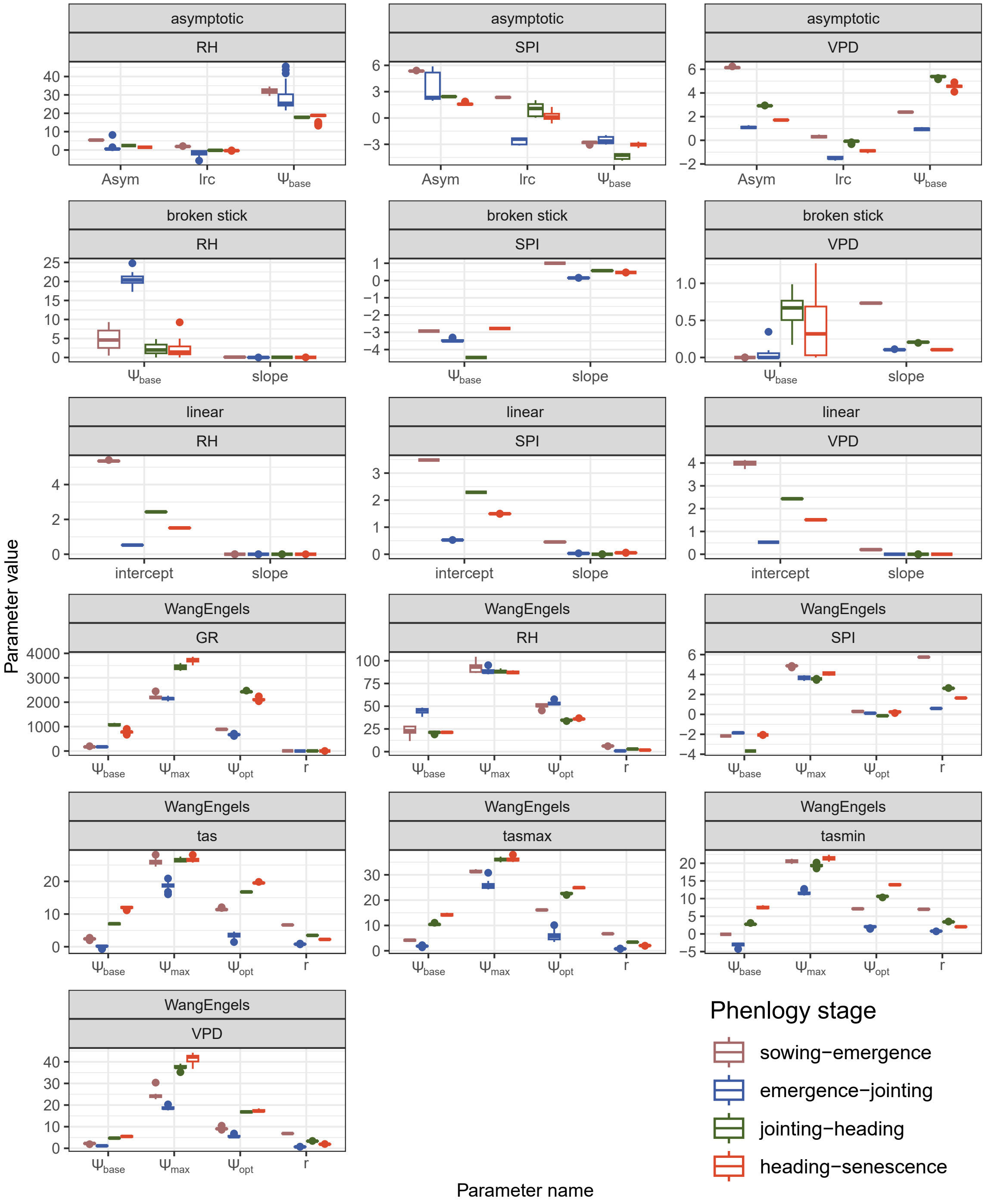

Figure A.4: Overview of the optimized parameters for all DRC curves for the phenology phases (sowing-emergence, emergence-jointing, jointing-heading and heading-senescence - different colors) for all covariates. Box-plots show the 20 iteration ensemble done in the optimization step. The different panels represent a DRC type per environmental covariate. On the x-axis the different parameters as listed, on the y-axis the corresponding optimized values.

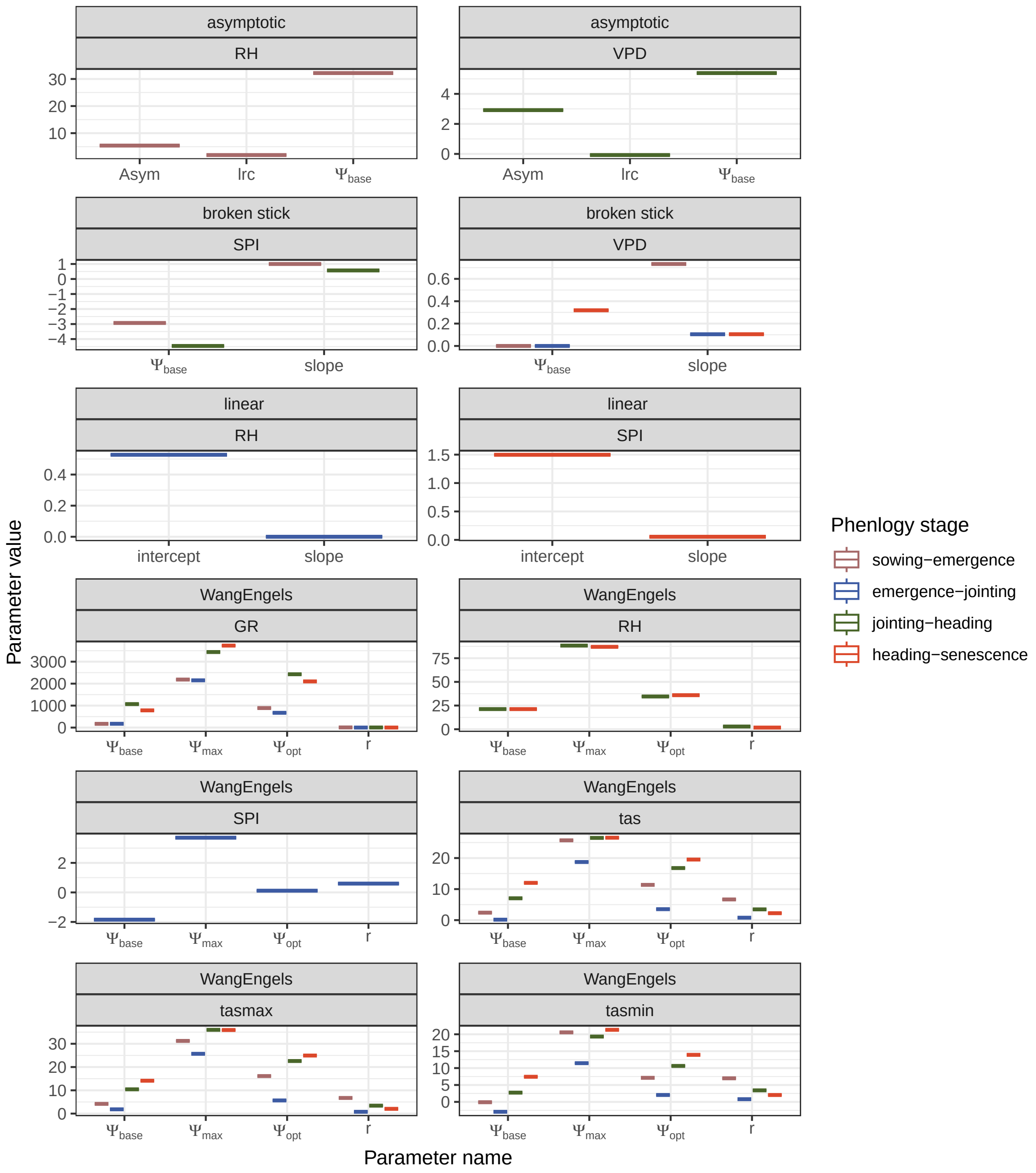

Figure A.5: Overview of the optimized parameters for all selected DRC curves per phenology phase (sowing-emergence, emergence-jointing, jointing-heading and heading-senescence - different colors) and covariate. The different panels represent a DRC type per environmental covariate. On the x-axis the different parameters are listed, on the y-axis the corresponding optimized values.

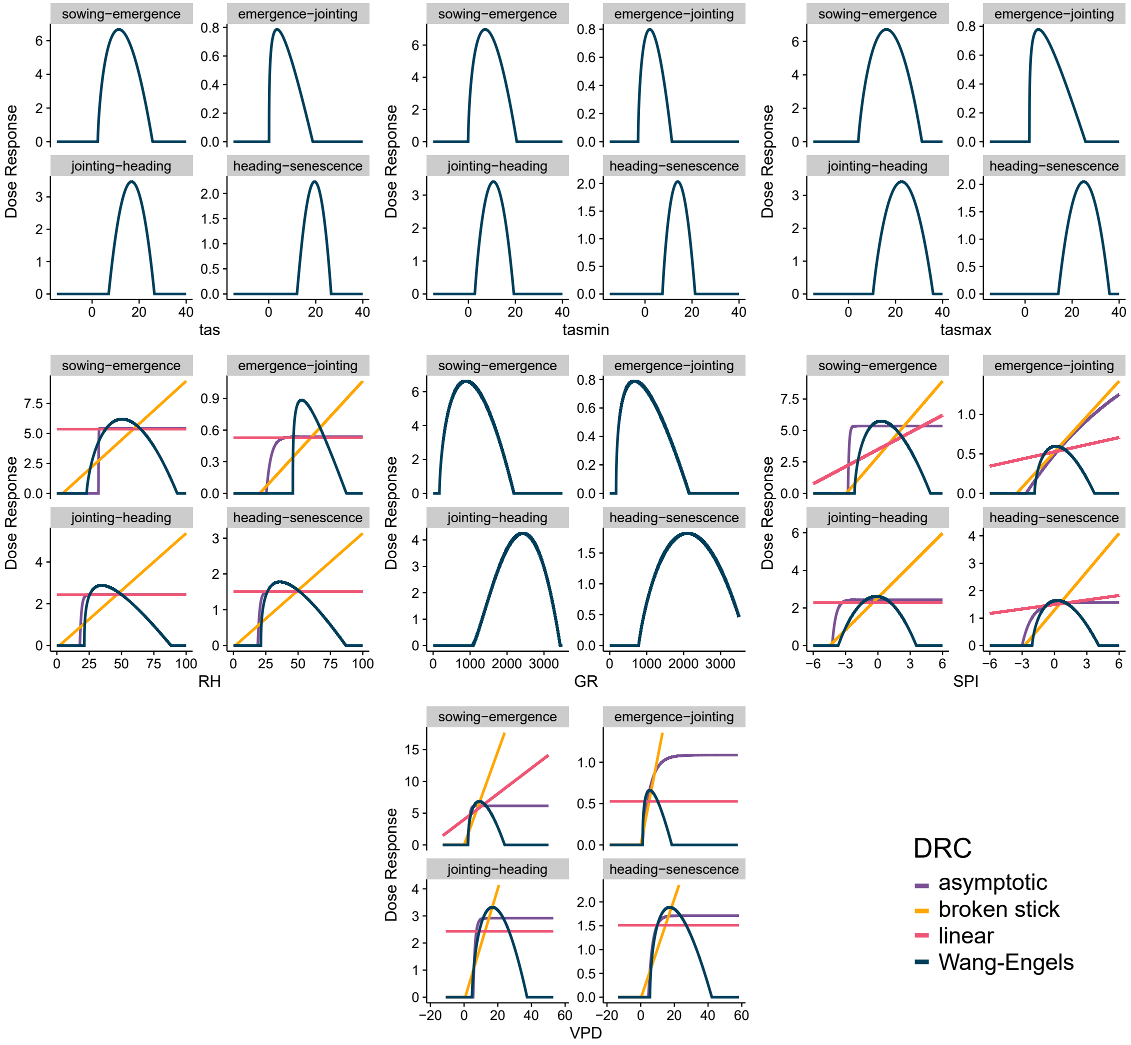

Figure A.6: Overview of the fitted DRC curves for the phenology phases (sowing-emergence, emergence-jointing, jointing-heading and heading-senescence) in the different panels per environmental variable. The colors represent the four different DRC curves, on the x-axis are the environmental covariates (tas, tasmin, tasmax, RH, GR, SPI and VPD), on the y-axis are the corresponding dose response values (see for further values Table A.3).

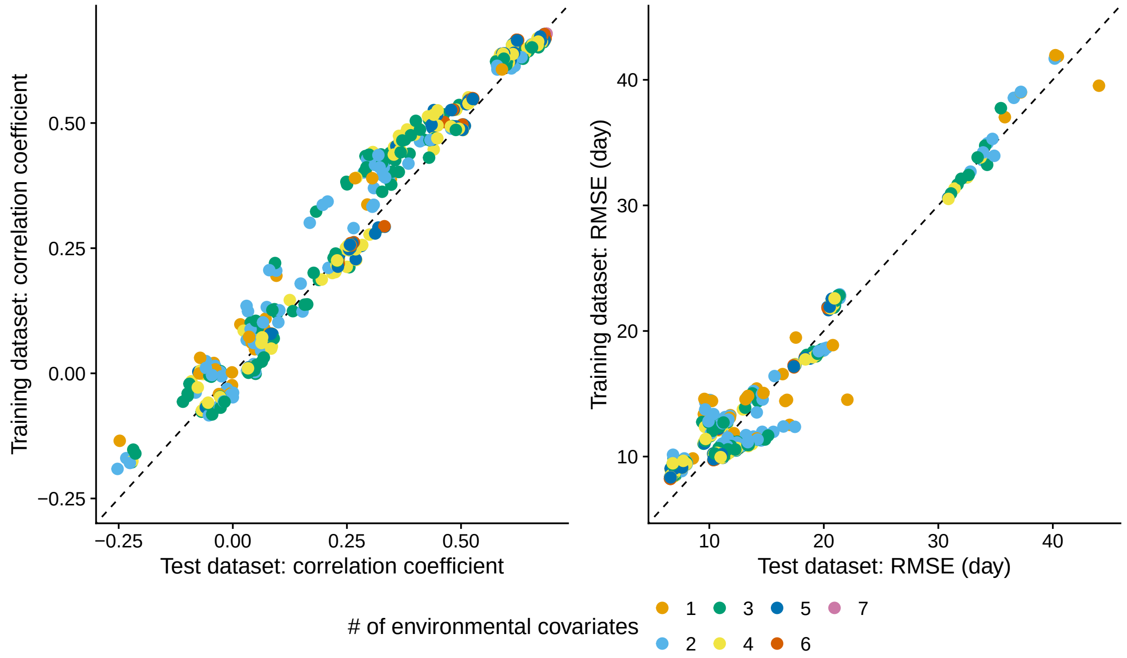

Figure A.7: Relation between the correlation coefficient (left) and the root mean squared error (RMSE; right) of all 508 fitted models on the test dataset (x-axis) against the training dataset (y-axis) to evaluate potential overfitting issues. The colors mark the different model complexities used, meaning the number of environmental covariates used for each model.

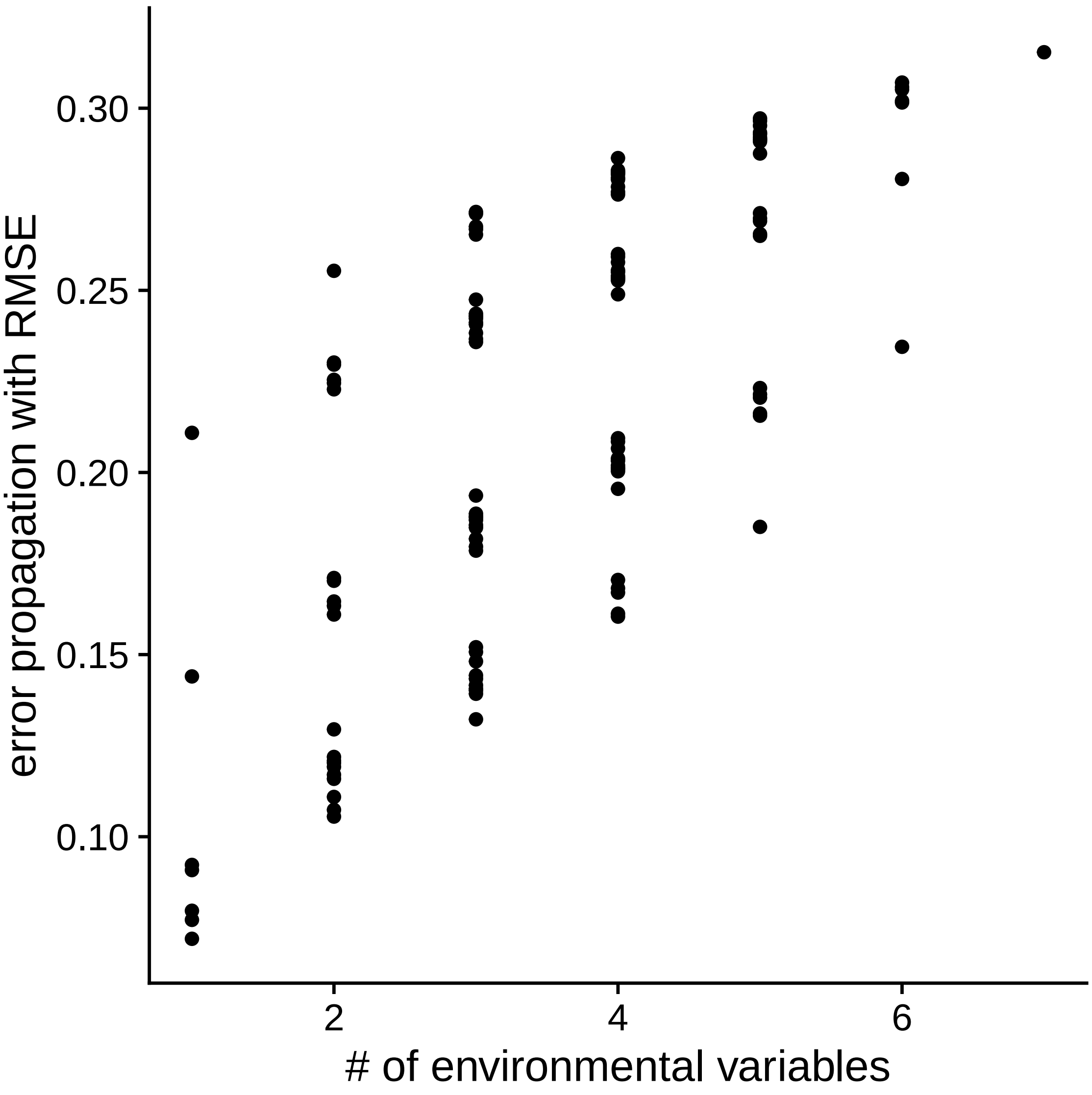

Figure A.8: Uncertainty propagation of all used environmental covariate combinations, from one up to seven environmental covaraites (x-axis; 127 combinations). The min/max standardized RMSE value between the observation and climate scenario data of all stations during the reference period (1981-2010) have been used to calculate the uncertainty propagation (y-axis). Higher values indicate a higher propagated uncertainty of the specific combination.

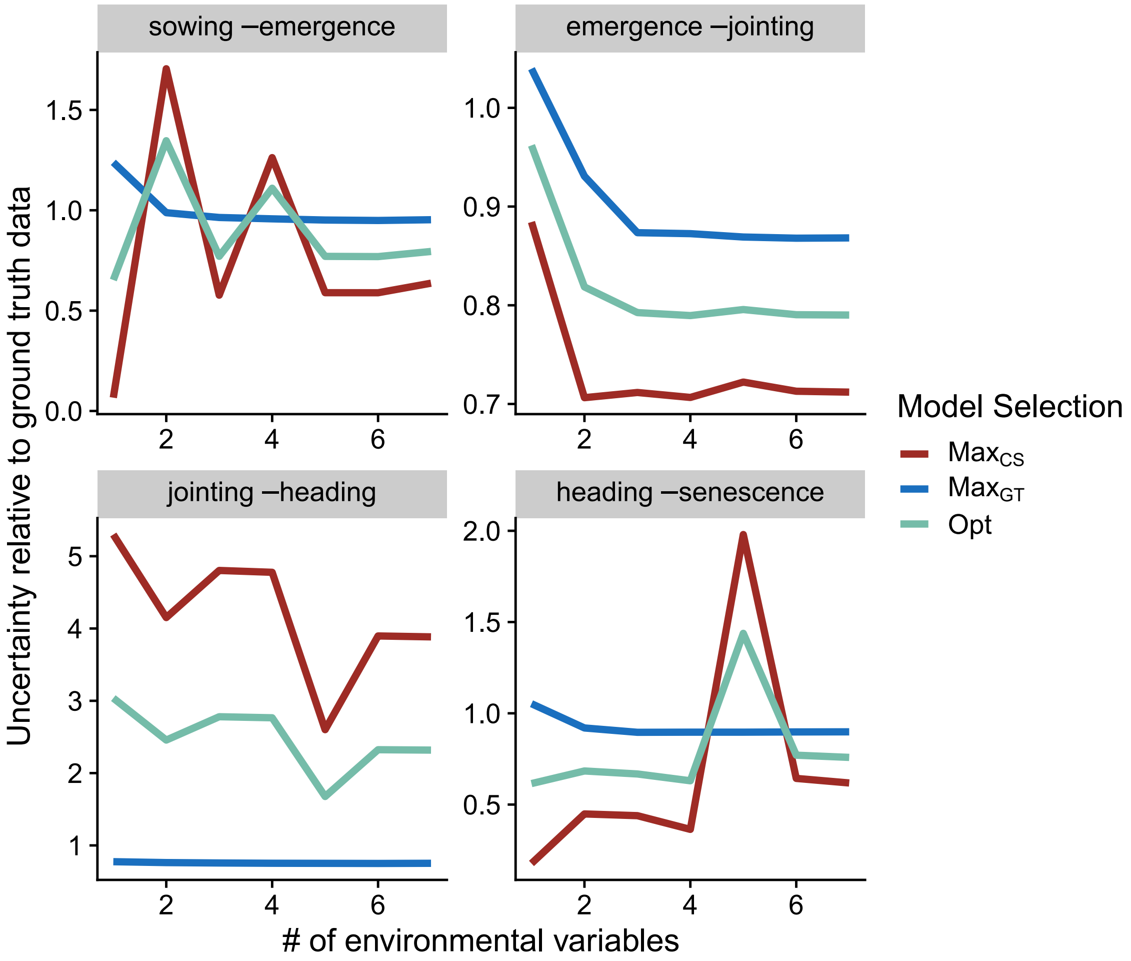

Figure A.9: Relative uncertainty compared to the ground truth derived standard deviation from the DWD phenology observation data of the model is shown on the y-axis, where the x-axis represents the model complexity. The relative score derive from the model RMSE values and have been standardized by dividing through the ground truth standard deviation to result in comparable relative values. Each panel represents one phenological phase. The red line represents the Max_{CS}, blue the Max_{GT} and green the Opt model selection.

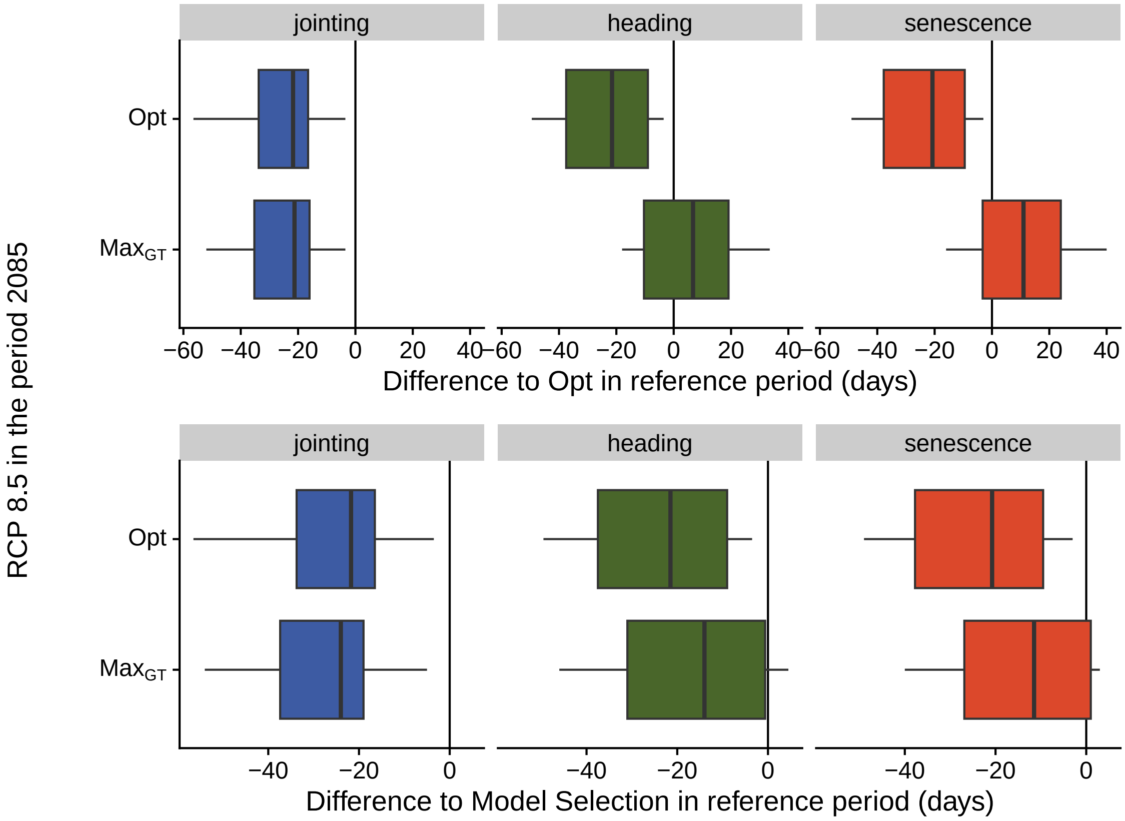

Figure A.10: Comparison between the reference period (1981-2010) and the 2085 period (2070-2099) of the RCP 8.5 scenario. The upper row shows the difference between the *Opt* model selection in the reference period to the 2085 period in days (x-axis) per phenological stage and all model selections (y-axis). The lower panel shows the difference between the corresponding model selections in the reference to the 2085 period (See also Table A.8).

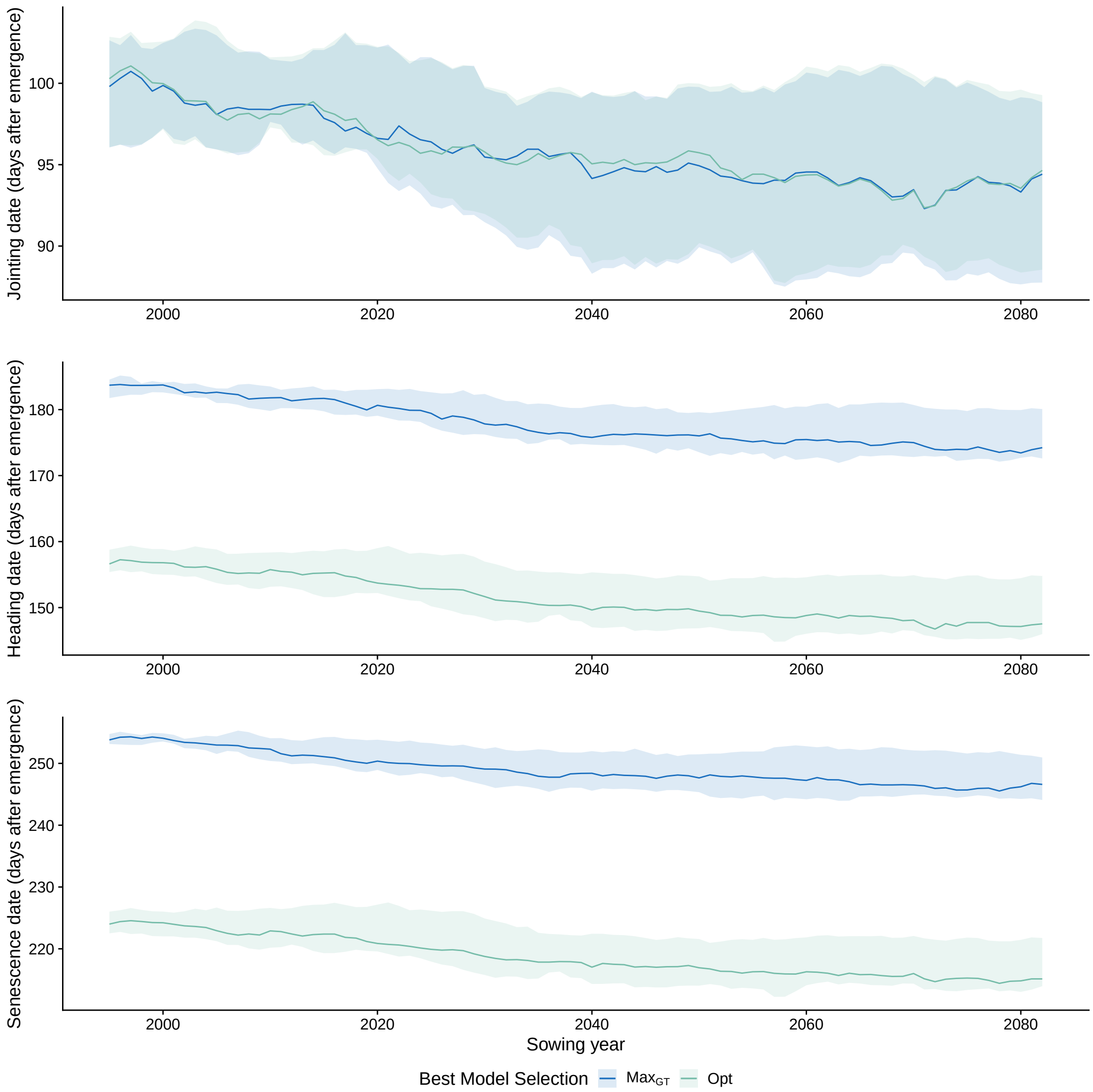

Figure A.11: Projection of jointing, heading and senescence date until 2100 with the above selected as best models for the RCP 2.6 scenario at the station REH (Zurich Reckenholz). The x-axis shows the sowing years the line shows the median, the shape the 5 and 95% quantile of the applied modelchains.

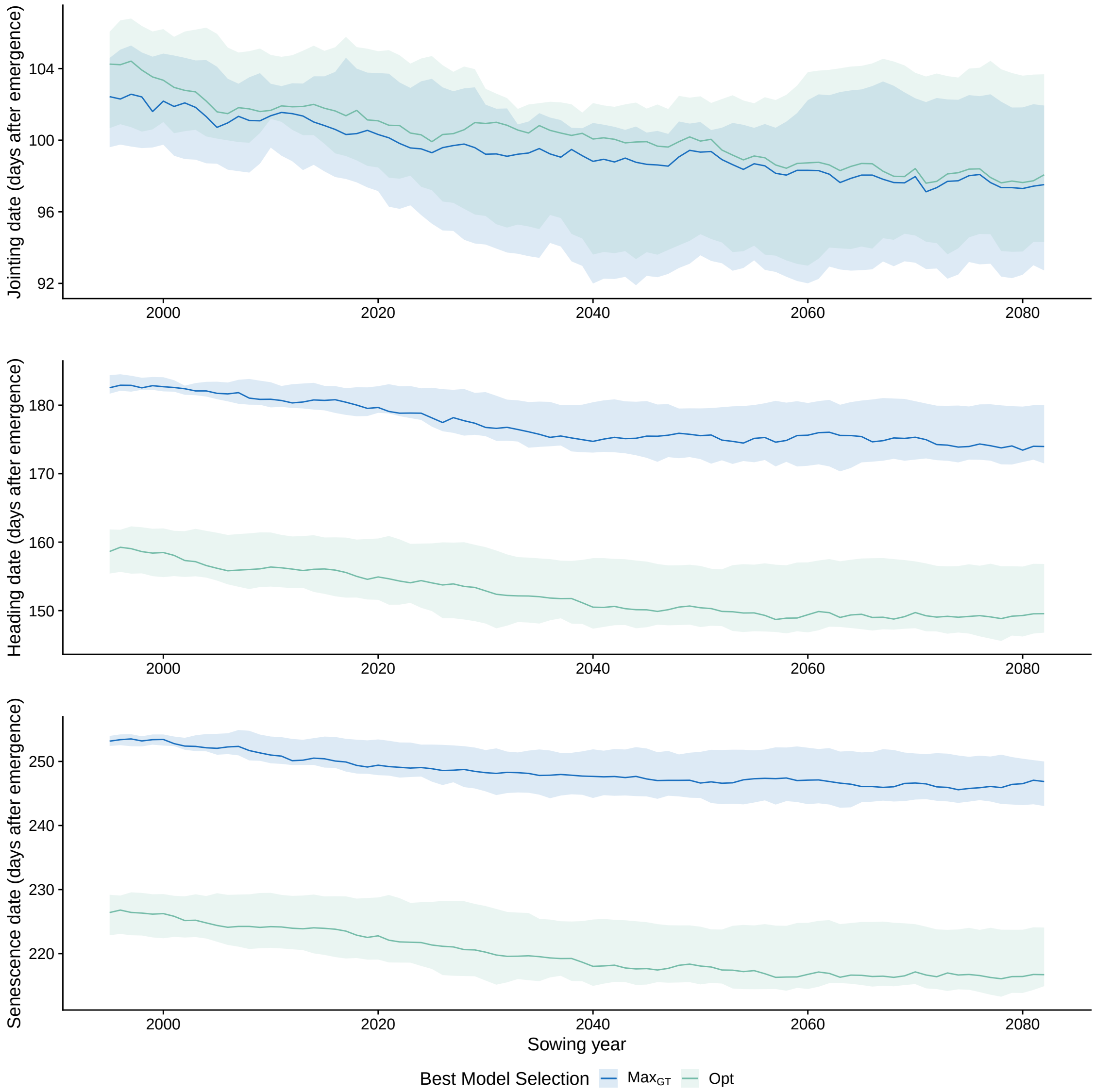

Figure A.12: Projection of jointing, heading and senescence date until 2100 with the above selected as best models for the RCP 2.6 scenario at the station SHA (Schaffhausen).The x-axis shows the sowing years the line shows the median, the shape the 5 and 95% quantile of the applied modelchains.

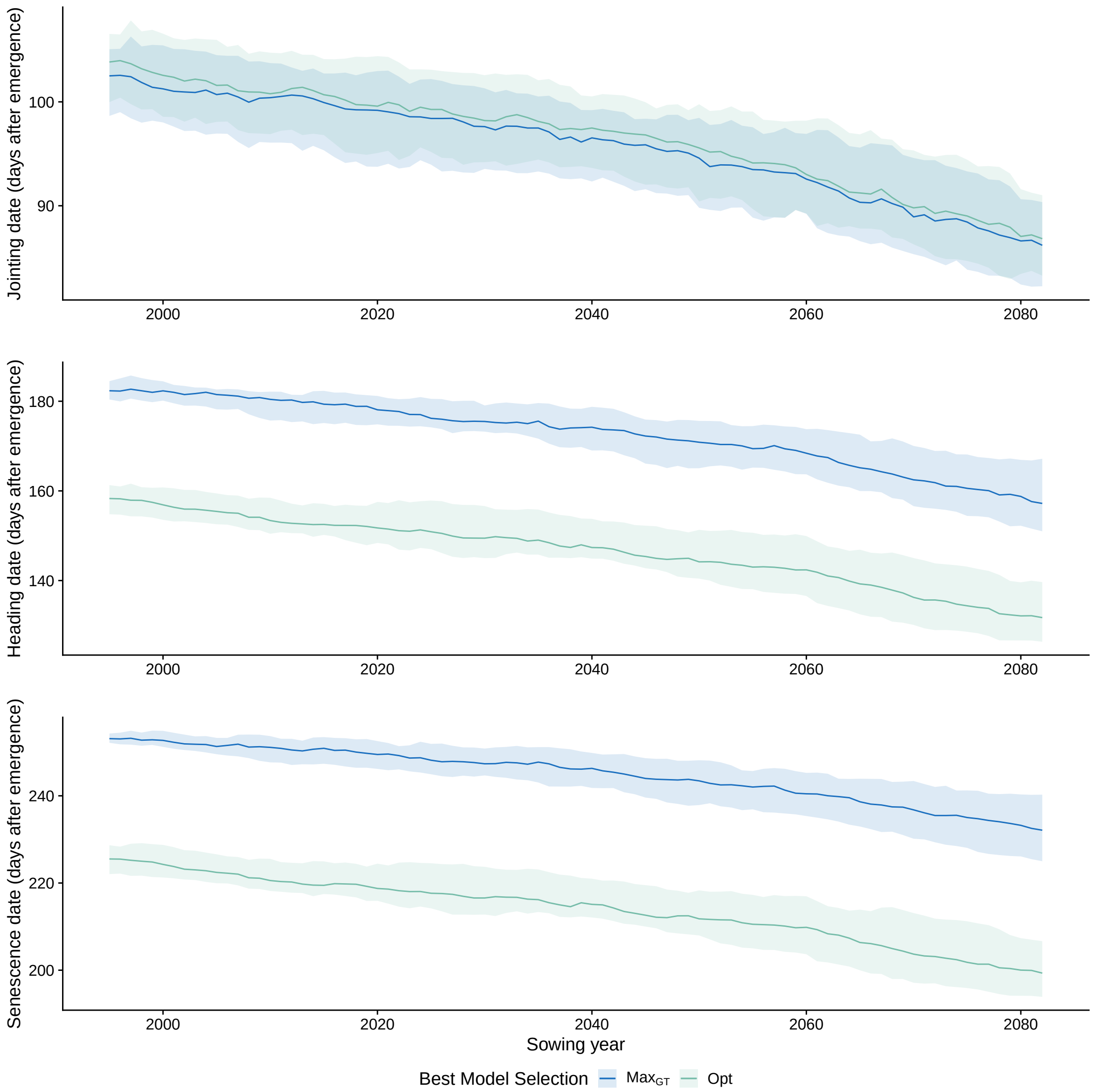

Figure A.13: Projection of jointing, heading and senescence date until 2100 with the above selected as best models for the RCP 8.5 scenario at the station SHA (Schaffhausen).The x-axis shows the sowing years the line shows the median, the shape the 5 and 95% quantile of the applied modelchains.

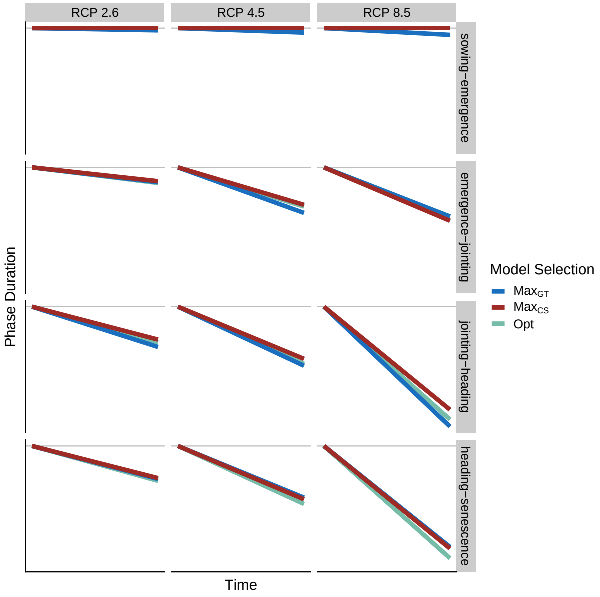

Figure A.14: Trend line over time (x-axis) per scenario (columns) per phenological phase (rows). For the station REH (Zürich Reckenholz).

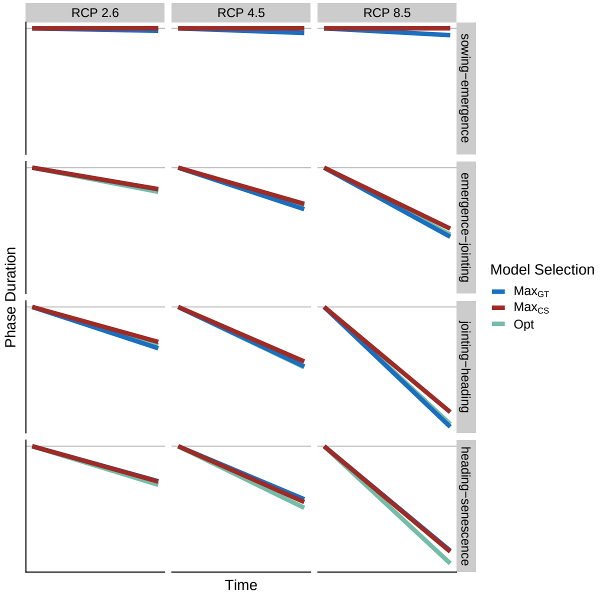

Figure A.15: Trend line over time (x-axis) per scenario (columns) per phenological phase (rows). For the station SHA (Schaffhausen).

A.2 Supplementary Tables

Table A.1: Correlation between JRC and DWD data of direct comparable environmental covariates: tas,tasmax,tasmin, GR and pr

environmental covariate	correlation coefficient
tas	0.99
tasmax	1
tasmin	0.98
GR	0.97
pr	0.9

Table A.2: Overview of the start parameter, lower and upper boundaries for the parameter estimation. For environment dependent parameters, the corresponding quantiles (Q) have been considered. For the environment independent parameters prior knowledge was used to determine the start values.

dose response curve	parameter name	lower	start	upper
linear	intercept	Q 0.01	Q 0.05	Q 0.95
linear	slope	0	0.05	0.5
broken stick	T_{base}	Q 0.05	Q 0.1	Q 0.6
broken stick	slope	0	0.05	0.5
asymptotic	Ψ_{base}	Q 0.05	Q 0.1	Q 0.4
asymptotic	lrc	-15	-1	1.5
asymptotic	asymptote	0	1/30	1
Wang-Engels	Ψ_{base}	Q 0.1	Q 0.25	Q 0.4
Wang-Engels	Ψ_{opt}	Q 0.6	Q 0.85	Q 0.98
Wang-Engels	Ψ_{max}	Q 0.7	Q 0.95	Q 0.99
Wang-Engels	r	1E-07	1	100

Table A.3: For each phenological phase and each environmental covariate the according to best correlation coefficient selected DRC curve with the corresponding correlation, RMSE (days) and MAE (days) value. All values derived from the training dataset.

Phenological phase	Environmental covariate	DRC	cor	RMSE	MAE
sowing-emergence	GR	Wang-Engels	0.067	8.981	3.025
sowing-emergence	RH	asymptotic	0.027	6.818	2.143
sowing-emergence	SPI	broken stick	0.083	24.338	20.266
sowing-emergence	tas	Wang-Engels	0.18	9.592	3.042
sowing-emergence	tasmax	Wang-Engels	0.173	9.413	2.974
sowing-emergence	tasmin	Wang-Engels	0.201	9.547	3.292
sowing-emergence	VPD	broken stick	0.161	12.051	6.522
emergence-jointing	GR	Wang-Engels	-0.254	42.379	8.614
emergence-jointing	RH	linear	0.093	21.868	2.821
emergence-jointing	SPI	Wang-Engels	0.098	44.393	12.386
emergence-jointing	tas	Wang-Engels	-0.072	54.823	20.035
emergence-jointing	tasmax	Wang-Engels	-0.094	50.151	14.025
emergence-jointing	tasmin	Wang-Engels	-0.053	49.976	18.078
emergence-jointing	VPD	broken stick	0.631	17.882	4.429
jointing-heading	GR	Wang-Engels	0.407	13.851	4.831
jointing-heading	RH	Wang-Engels	0.089	15.353	5.529
jointing-heading	SPI	broken stick	0.082	18.798	8.671
jointing-heading	tas	Wang-Engels	0.662	11.308	3.675
jointing-heading	tasmax	Wang-Engels	0.62	11.793	3.672
jointing-heading	tasmin	Wang-Engels	0.614	11.768	3.832
jointing-heading	VPD	asymptotic	0.401	13.617	4.67
heading-senescence	GR	Wang-Engels	0.083	13.715	3.12
heading-senescence	RH	Wang-Engels	0.159	13.585	2.715
heading-senescence	SPI	linear	-0.003	11.913	2.204
heading-senescence	tas	Wang-Engels	0.434	11.591	2.047
heading-senescence	tasmax	Wang-Engels	0.419	11.065	2.013
heading-senescence	tasmin	Wang-Engels	0.295	12.287	1.228
heading-senescence	VPD	broken stick	0.351	17.109	5.53

Table A.4: Shown for each phenological phase the selected best model (Max_{GT}) according to lowest RMSE. In the table are additionally shown the corresponding correlation coefficients and MAE values. Furthermore, the number of used environmental covariates and which environmental covariates have been chosen.

phenology phase	correlation coefficient	RMSE	MAE	# of used environmental variables	environmental variables
sowing-emergence	0.265	6.56	3.437	6	tas, tasmin, tasmax, RH, GR, SPI
emergence-jointing	0.625	17.358	3.864	6	tasmin, tasmax, RH, GR, SPI, VPD
jointing-heading	0.678	10.714	4.106	6	tasmin, tasmax, RH, GR, SPI, VPD
heading-senescence	0.506	10.337	2.537	3	tas, GR, SPI

Table A.5: Information of the used MeteoSwiss stations for this study

Station name	Station abbreviation	Latitude	Longitude	Altitude (m.a.s.l)
Aadorf / Tänikon	TAE	47.48	8.9049	539
Aigle	AIG	46.327	6.9244	381
Altdorf	ALT	46.887	8.6218	438
Bad Ragaz	RAG	47.017	9.5026	496
Basel / Binningen	BAS	47.541	7.5836	316
Bern / Zollikofen	BER	46.991	7.464	552
Buchs / Aarau	BUS	47.384	8.0794	386
Chur	CHU	46.87	9.5305	556
Elm	ELM	46.924	9.1753	958
Fahy	FAH	47.424	6.9411	596
Genève / Cointrin	GVE	46.248	6.1277	410
Glarus	GLA	47.035	9.0669	516
Güttingen	GUT	47.602	9.2794	440
Interlaken	INT	46.672	7.8701	577
Locarno / Monti	OTL	46.173	8.7874	366
Lugano	LUG	46.004	8.9603	273
Luzern	LUZ	47.036	8.301	454
Magadino / Cadenazzo	MAG	46.16	8.9337	203
Meiringen	MER	46.732	8.1692	588
Neuchatel	NEU	47	6.9533	485
Nyon / Changins	CGI	46.401	6.2278	455
Payerne	PAY	46.812	6.9424	490
Piotta	PIO	46.515	8.6881	990
Pully	PUY	46.512	6.6674	455
Rünenberg	RUE	47.435	7.8793	611
Schaffhausen	SHA	47.69	8.6201	438
Sion	SIO	46.219	7.3302	482
St. Gallen	STG	47.425	9.3985	775
Stabio	SBO	45.843	8.9324	353
Vaduz	VAD	47.127	9.5175	457
Visp	VIS	46.303	7.8429	639
Wynau	WYN	47.255	7.7874	422
Wädenswil	WAE	47.221	8.6777	485
Zürich / Affoltern	REH	47.428	8.5179	443
Zürich / Fluntern	SMA	47.378	8.5657	555
Zürich / Kloten	KLO	47.48	8.5359	426

Table A.6: Fixed start dates per year for the phenology phases applied to the CH2018 data for validation purposes. The dates are derived from the median values of the available observation data.

Phenology phase	Start date
sowing-emergence	15. October
emergence-jointing	27. October
jointing-heading	5. May
heading-senescence	9. June

Table A.7: Standard deviation (in days) of the phenological phase duration of the DWD derived ground truth data.

Phenology phase	standard deviation
sowing-emergence	6.91
emergence-jointing	20.00
jointing-heading	14.27
heading-senescence	11.54

Table A.8: Comparison between the reference period (1981-2010) and the 2085 period (2070-2099) of the RCP 8.5 scenario. The Difference was calculated between the Opt model selection in the reference period to the 2085 period in days per phenological stage and all model selections. Furthermore, between the corresponding model selections in the reference to the 2085 period (See also Figure A.10).

Phenological Phase	Model Selection	Difference to Opt in reference period (days)	Difference to corresponding model selection in reference period (days)
jointing	Max_{GT}	-21.25	-24
	Max_{CS}	-18.5	-20
	Opt	-21.75	-21.5
heading	Max_{GT}	6.75	-14
	Max_{CS}	-19.5	-20.75
	Opt	-21.5	-21.5
senescence	Max_{GT}	11	-11.5
	Max_{CS}	-19.5	-19.5
	Opt	-20.75	-20.75

Table A.9: Heading date scorings from 14 environments where the GABI wheat panel was grown from the Gogna et al. 2022 and our own (Zurich) research stations. For each environment the first and the last genotype that reached heading is shown, as well as the range in days between the first and the last genotype. To reduce field heterogeneity effects, genotype BLUEs have been used.

Location	Year	First genotype BLUE (DOY)	Last genotype BLUE (DOY)	range in days	# of genotypes
Wohl (Germany)	2010	150.5	166.6	16.1	381
Seligenstadt (Germany)	2010	150	163	13	381
Saultain (France)	2010	142.8	159.6	16.8	381
Janville (France)	2010	142.1	161.1	19	381
Andelu (France)	2010	139.3	157.3	18	381
Wohl (Germany)	2009	135.1	155	19.9	381
Seligenstadt (Germany)	2009	142.5	158	15.5	381
Andelu (France)	2009	134.5	164.1	29.6	381
Zurich (Switzerland)	2022	138.5	154.3	15.8	373
Zurich (Switzerland)	2021	154.8	163.6	8.8	346
Zurich (Switzerland)	2019	152.1	165.7	13.6	78
Zurich (Switzerland)	2018	135.1	149.7	14.6	353
Zurich (Switzerland)	2017	145	157	12	352
Zurich (Switzerland)	2016	146.8	158.8	12	335
